# Medicaid-covered health care visits during the postpartum year: Variation by enrollee characteristics and state

**DOI:** 10.1093/haschl/qxaf019

**Published:** 2025-01-30

**Authors:** Laura Barrie Smith, Claire O’Brien, Keqin Wei, Timothy A Waidmann, Genevieve M Kenney

**Affiliations:** Urban Institute, Health Policy Division, Washington, DC 20024, United States; Urban Institute, Health Policy Division, Washington, DC 20024, United States; Urban Institute, Health Policy Division, Washington, DC 20024, United States; Urban Institute, Health Policy Division, Washington, DC 20024, United States; Urban Institute, Health Policy Division, Washington, DC 20024, United States

**Keywords:** Medicaid, postpartum, pregnancy, postpartum extensions, maternal health

## Abstract

Extending pregnancy-related Medicaid eligibility from 60 days to 12 months postpartum represents an important opportunity to reduce maternal mortality and racial inequities in maternal health outcomes. However, patterns of health care service use after 60 days postpartum among Medicaid enrollees are not well understood. We use Medicaid claims data representing Medicaid-covered live births in 46 states in 2018 to examine outpatient visits during the postpartum year. We find that more than three-quarters of enrollees with full-year Medicaid coverage have at least one outpatient visit between 61 days and 12 months postpartum. The share of enrollees with visits varies from 51.5% to 88.0% across states and is higher among enrollees with diagnosed physical or mental/behavioral health conditions or pregnancy/delivery complications. We also find that visits including mental/behavioral health care are more common for non-Hispanic white enrollees than non-Hispanic Black and Hispanic enrollees and for rural enrollees than urban enrollees during the postpartum year, controlling for other characteristics. These findings suggest that many Medicaid enrollees who maintain Medicaid coverage beyond 60 days postpartum will receive outpatient care but also suggest that there may be inequities in receipt of postpartum health care across and within states.

## Introduction

The United States has a concerningly high maternal mortality rate, and many maternal complications and deaths occur after delivery and up to 12 months postpartum.^1,2^ There are also considerable racial disparities in maternal health outcomes; for example, pregnancy-related mortality rates are nearly three times higher for Black compared to white individuals.^3^ In response to this maternal health crisis, Sections 9812 and 9822 of the American Rescue Plan (ARP) of 2021 gave states a new option to extend pregnancy-related eligibility for Medicaid coverage from 60 days to 12 months postpartum.^4,5^ Given that over 40% of all US births and nearly 65% of US births to Black individuals are covered by Medicaid or the Children's Health Insurance Program (CHIP), the postpartum extensions represent an opportunity for states to improve health insurance coverage, affordability, and equity in the postpartum period.^6^ As of December 2024, 47 states including the District of Columbia (DC) have implemented 12-month Medicaid/CHIP postpartum extensions.^7^

Prior to 12-month postpartum Medicaid extensions, pregnancy-related Medicaid coverage expired 60 days after pregnancy in all states. After this 60-day period, Medicaid eligibility pathways varied considerably by state, with enrollees in states that adopted ACA Medicaid expansions more likely to maintain coverage due to higher income eligibility thresholds. For example, in 2018 in expansion states, the median income eligibility threshold for non-pregnant parents was 138% of the Federal Poverty Level (FPL) compared to a median of 50% FPL in non-expansion states.^8-10^

The potential loss of Medicaid coverage after 60 days postpartum puts new parents at risk of experiencing gaps in health care access and affordability, particularly if they become uninsured. Prior research finds many uninsured new parents have physical or mental health conditions that call for ongoing care in the postpartum period.^11,12^ Furthermore, nearly one-quarter of uninsured new parents face unmet needs for health care, and over half are worried about medical bills.^12^

The extent to which the recently adopted 12-month Medicaid postpartum extensions have improved access to care after pregnancy has not yet been documented. More generally, patterns of health care service use during the postpartum year among Medicaid enrollees are not well understood. It is important to quantify health care use during the postpartum year to understand patterns of care during this critical period of time in people's lives and to inform potential impacts of the 12-month postpartum extensions and strategies to maximize their impacts.

Prior research on postpartum service use has primarily focused on receipt of a single postpartum evaluation visit as recommended by the American College of Obstetricians and Gynecologists.^13^ Estimated rates of receiving this postpartum evaluation visit vary widely, from approximately 25% to over 95%.^14^ Several studies find postpartum evaluation visit rates are lower for Medicaid compared to privately insured populations,^14-16^ and 2019 Centers for Medicare and Medicaid Services (CMS) data from 39 states suggest that in the median state, 72% of Medicaid enrollees had a postpartum evaluation visit.^17^

Lower postpartum evaluation visit rates among Medicaid compared to privately insured individuals may be due in part to the historic loss of pregnancy-related Medicaid at 60 days postpartum. Prior studies examining Medicaid-covered service use during the later postpartum period have found retaining Medicaid coverage after 60 days to be associated with higher levels of postpartum, preventive, outpatient, and mental/behavioral health services and treatment.^18-20^ However, these studies have focused on enrollees from 1 or 2 states or a single managed care plan.

In this study, we provide new evidence on the share of Medicaid-covered health care visits during the postpartum year, focusing on the period of time between 61 days and 12 months postpartum. We examine the share of enrollees with any visit and the share of enrollees with visits for different types of care, including postpartum evaluation, preventive/well care, contraceptive management, mental/behavioral health care, and care for other acute or chronic illness. We use 2018-2019 Medicaid claims and encounter data representing all Medicaid-covered deliveries in 46 US states (including DC). We also assess differences in postpartum visits by age, race/ethnicity, rurality, eligibility pathway, physical and mental/behavioral health conditions, pregnancy and delivery complications, and state. We stratify our analyses by state Medicaid expansion status, since prior to the postpartum extensions, eligibility for Medicaid beyond the 60-day postpartum period was much more limited in non-expansion states.^8^

While data on the 12-month postpartum extensions implemented under ARP are not yet available, this study is the first to our knowledge to use a near-national Medicaid data set to document patterns of outpatient care during the postpartum year among Medicaid enrollees. This analysis represents an important first step in assessing the extent to which the 12-month extensions could help meet the health care needs of postpartum individuals and reduce inequities in maternal health outcomes, which can inform evidence-based policies in Medicaid for the postpartum period.

## Study data and methods

### Data and sample

We used Medicaid claims and enrollment data from the Transformed Medicaid Statistical Information System (T-MSIS) Analytic Files (TAF) from 2018 to 2019. These data comprise records of all Medicaid-covered services and enrollee characteristics including eligibility category, days enrolled, and basic demographic characteristics.

Our analytic sample included individuals with a Medicaid-covered live birth in 2018 in 46 states (including DC), following best practices from prior literature to identify live births.^21,22^ We excluded Florida, Minnesota, Massachusetts, New Jersey, and Rhode Island due to data quality concerns (see Appendix 1 for details).^23^ We excluded the three percent of enrollees who moved to a different state during the 60 days following their delivery and the less than one percent of enrollees dually enrolled in Medicare since their postpartum services would likely not appear in the Medicaid data. We identified enrollees as having 12 months of continuous enrollment if they were enrolled in full-benefit Medicaid coverage in the same state each month for 12 months following delivery (Appendix 2 compares the characteristics of those who had 12 months of continuous enrollment and those who did not).

### Variables

The main outcome of interest was the occurrence of any outpatient health care visit, which excludes care provided in an inpatient, emergency department, or urgent care setting. We additionally identified the types of care received within visits based on diagnosis and/or procedure codes: postpartum evaluation, preventive/well care, contraceptive management, mental/behavioral health care, and care for other acute or chronic illness (see Appendix 3 for details on these definitions). We note that a single visit might include more than one of these types of care (though this is the case for less than 3.5% of all visits).

We assessed the share of enrollees with at least one visit during two postpartum time periods, defined to be consistent with Medicaid eligibility rules^5,24^: the initial 60 days (the day following delivery through the end of the month of the 60th day following delivery) and 61 days to 12 months (the first day of the month following the 60-day period through the end of the 12th month following delivery).

We defined age groups based on age in years at the time of delivery (< 19, 19 to 24, 25 to 29, 30 to 34, and 35+); urban vs rural based on zip code of residence^25^; and Medicaid eligibility category during the 12th month postpartum (pregnancy, parent, adult expansion, child, Supplemental Security Income (SSI), transitional Medical Assistance, other, multiple/missing). We defined race/ethnicity based on the categories available in the TAF. Since there are well-known concerns with the quality of the race/ethnicity variable in the TAF, we compared the share of births by race/ethnicity in the TAF to the share of Medicaid-covered births by race/ethnicity according to 2018 natality data from the Centers for Disease Control and Prevention (CDC). For our primary race/ethnicity analyses, we included 20 states where the share of non-Hispanic Black (hereafter, Black), Hispanic, and non-Hispanic white (hereafter, white) deliveries from the TAF were within 10 percentage points of the comparable CDC estimates and where less than 20% of enrollees had a missing value in the TAF. We conducted analyses on American Indian/Alaska Native, Asian, and Hawaiian/Pacific Islander populations on smaller subsets of states with sufficient data quality (Appendix 4).

We also created indicators for underlying (non-pregnancy-related) chronic conditions (diabetes, hypertension, and obesity), preterm birth, cesarean section (c-section), other pregnancy and labor/delivery complications, and mental/behavioral health conditions diagnosed prior to or during the delivery hospitalization (Appendix 3).^18^ We did not control for diagnoses that emerged during the postpartum period, as these would be correlated with having visits.

### Analysis

We calculated the share of individuals with Medicaid-covered births in 2018 who had at least one Medicaid-covered outpatient visit within 60 days postpartum and between 61 days and 12 months postpartum. We assessed the share with any type of visit and the share with a visit that included each type of care. We stratified the analyses by state Medicaid expansion status since a significantly larger share of enrollees disenrolled between 60 days and 12 months postpartum in non-expansion states (Appendix 2)—-and those who maintained coverage in non-expansion states would be expected to have lower incomes, on average, compared with those maintaining coverage in expansion states.

We then assessed the share of enrollees with at least one visit of any type and visits by type of care by demographic characteristics, enrollment categories, and presence of preexisting physical and mental/behavioral conditions and pregnancy/delivery complications. Because these characteristics are correlated with each other, we estimated associations before and after adjusting for other observed characteristics (see Appendix 5 for details). We also examined state variation in the share of enrollees with at least one visit between 61 days and 12 months postpartum, before and after adjusting for observed individual characteristics since the composition of the continuously enrolled populations varies across states. In supplementary analyses, we repeated the analyses focused on enrollees in ACA expansion states enrolled in Medicaid through the expansion pathway and found similar patterns to the main results (see Appendix 6).

### Limitations

While the Medicaid TAF data are designed to be uniform across states and provide a comprehensive record of Medicaid-covered services, they have some quality issues that vary by state. We excluded 5 states with known data quality concerns related to the volume of their outpatient claims.^23^ We conducted sensitivity analyses excluding 9 additional states for which the number of Medicaid-covered births we identified varied by more than 20% from estimates published by states or the CDC and found that the results were similar to our main results. For comparisons by race/ethnicity, we excluded 26 states for having poor race and ethnicity data for the three largest race/ethnicity groups (Black, Hispanic, and white), so the patterns we analyze by race/ethnicity may not be representative of patterns in all US states. We also conducted analyses on smaller race/ethnicity groups, but these comparisons were limited to even fewer states and must also be interpreted with caution (Appendix 4).

Second, Medicaid reimbursement for maternity care is often provided through a bundled payment that covers all prenatal and labor and delivery services and, in some cases, a routine postpartum visit within 60 days.^26,27,28^ When bundled payments are used, postpartum evaluation visits may be unobservable in the TAF.^29^ Therefore, in states that frequently use bundled payments for maternity care, our analysis may underestimate the share of individuals with a postpartum evaluation visit within 60 days (see Appendix 7). However, the primary focus of this analysis is on the time period between 61 days and 12 months postpartum when postpartum evaluation visits would not be part of a maternity care bundle.

Third, underlying physical and mental/behavioral health conditions were likely undercounted in our study population since they only reflect diagnoses recorded on claims files. Moreover, we did not have access to 2017 data. Sensitivity analyses limited to births in September through December 2018 (for which we have a minimum 9-month lookback period) showed similar associations between the conditions and visits compared with the main analysis (Appendix 8).

## Study results

There were 1 453 767 Medicaid enrollees in 46 states (including DC) with live births in 2018. Approximately 53.0% of the study population had 12 months of continuous enrollment following delivery, including 69.4% of the 861 757 expansion state enrollees and 29.2% of the 592 010 non-expansion state enrollees (Appendix 2).

**Figure 1.**
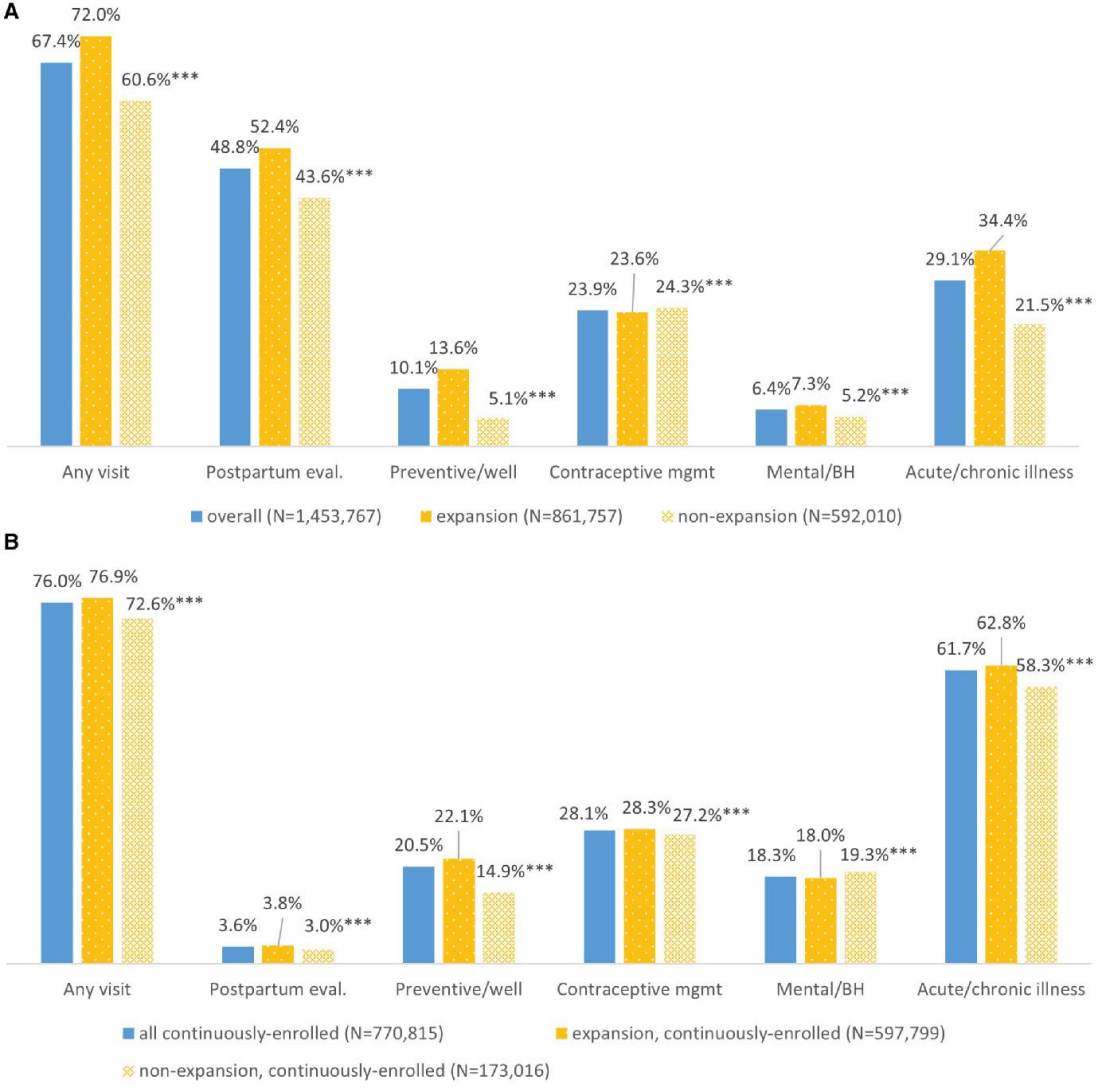
Share of Medicaid enrollees with at least one outpatient visit within 60 days postpartum and between 61 days and 12 months postpartum, among those with continuous enrollment, 2018 (Panel A: Share of enrollees with at least one visit within 60 days postpartum, among those with 60-day continuous enrollment (*N* = 1 453 767); Panel B: Share of enrollees with at least one visit between 61 days and 12 months postpartum, among those with 12 months continuous enrollment (*N* = 770 815)). Source/Notes: SOURCE 2018-2019 Transformed Medicaid Statistical Information System Analytic Files from 45 states plus DC. NOTES Shares by type of care sum to more than the share with any visit because enrollees may have more than one visit and a small number (3.3%) of visits include more than one type of care. Sample includes enrollees with Medicaid-covered births in 2018 (see Appendix 1 for details). State Medicaid expansion status is as of January 2018. In expansion states, 69.4% of enrollees had continuous enrollment for 12 months postpartum, compared with 29.2% of enrollees in non-expansion states. Appendix 10 shows the share of enrollees with outpatient visits within 60 days postpartum among those with continuous enrollment for 12 months. *** Estimate differs significantly between expansion and non-expansion states at the 0.01 level, using two-tailed tests. Eval.= evaluation. Mgmt = management. BH = behavioral health.

Figure 1 shows the share of enrollees with at least one visit within the first 60 days postpartum and between 61 days to 12 months postpartum, among those maintaining continuous enrollment. Within 60 days postpartum, 67.4% of enrollees had at least one visit. Approximately 48.8% of enrollees had a visit including postpartum evaluation, 10.1% had a visit including preventive/well care, 23.9% had a visit including contraceptive management, 6.4% had a visit including mental/behavioral health care, and 29.1% had a visit for acute or chronic illness (shares by types of care sum to more than the share with any type of visit since enrollees may have more than one visit). For all visit types except contraceptive management, these shares were significantly higher for enrollees in expansion compared to non-expansion states.

Between 61 days and 12 months postpartum, among all enrollees who maintained continuous Medicaid enrollment during this time, 76.0% had at least one visit; 3.6% had a visit including postpartum evaluation; 20.5% had a visit including preventive/well care; 28.1% had a visit including contraceptive management; 18.3% had a visit including mental/behavioral health care; and 61.7% had a visit for an acute or chronic illness (shares by types of care sum to more than the share with any type of visit since enrollees may have more than one visit). Except for mental/behavioral health care, these shares were significantly higher among those who were continuously enrolled in expansion compared to non-expansion states. Results for the full 12-month postpartum period as well as for visits within 60 days postpartum among enrollees maintaining coverage for the full 12 months postpartum follow similar patterns and are provided in Appendix 9 and 10.

Table 1 shows the share of enrollees with at least one visit within 60 days and between 61 days and 12 months postpartum, among those maintaining continuous enrollment during these time periods, by individual characteristics. While there are many differences by enrollee characteristics in the probability of having a visit, the characteristics that are associated with the largest differences include race/ethnicity, rural residence, eligibility pathway, and presence of underlying health conditions or pregnancy/delivery complications. In the 20 states where we were able to make comparisons by race/ethnicity, Black (74.9%) and Hispanic (73.9%) enrollees were less likely than white (79.1%) enrollees to have a visit between 61 days and 12 months. Adjusting for other observable characteristics, Black enrollees were 3.0% points (p.p.) less likely to have a visit and Hispanic enrollees were 2.9 p.p. less likely to have a visit than white enrollees (Appendix 5). Rural enrollees (79.3%) were more likely than urban enrollees (75.1%) to have a visit (adjusted difference, 4.2 p.p.; Appendix 5); enrollees eligible for Medicaid through SSI (84.5%) were more likely than enrollees eligible through the low-income parent pathway (76.3%) to have a visit (adjusted difference, 5.6 p.p.; Appendix 5); and enrollees with an underlying chronic health condition (79.3%), a preterm birth (78.9%), another pregnancy/delivery complication (77.9%), or a mental or behavioral health diagnosis during pregnancy (81.9%) were all more likely to have a visit compared to those without these conditions or complications (71.9%) (adjusted differences, 3.9 p.p., 2.8 p.p., 1.5 p.p., and 7.5 p.p., respectively). These patterns were generally similar in expansion and non-expansion states, with higher shares of enrollees having visits in expansion states for most groups. Results for the full 12-month postpartum period follow similar patterns (Appendix 11).

**Table 1.** Share of Medicaid enrollees with a visit within 60 days postpartum and between 61 days and 12 months postpartum, by individual characteristics, 2018.

	Births with 60 days of continuous enrollment postpartum (ie, all births in sample)		Births with 12 months of continuous enrollment postpartum					
	Visit within 60 days		Visit between 61 days and 12 months					
	All states in sample		All states in sample		Expansion states		Non-expansion states	
	*N*	Share (%)	*N*	Share (%)	*N*	Share (%)	*N*	Share (%)
All	1 453 767	67.4	770 815	76.0	597 799	76.9	173 016	72.6
Age in years								
Less than 19^^^	72 084	69.2	47 834	78.8	29 868	79.7	17 966	77.2
19-24	492 196	66.8***	262 905	75.6***	200 176	76.6***	62 729	72.3***
25-29	447 825	68.4***	237 006	75.7***	186 059	76.8***	50 947	71.7***
30-34	278 484	67.4***	142 748	75.9***	115 163	76.9***	27 585	72.0***
35 plus	163 178	65.6***	80 322	76.3***	66 533	77.0***	13 789	72.8***
Race/Ethnicity^c^								
White NH^^^	286 441	73.4	166 731	79.1	140 459	79.1	26 272	79.1
Hispanic	312 545	66.2***	120 409	73.9***	105 666	73.7***	14 743	74.8***
Black NH	153 342	71.3***	89 077	74.9***	72 112	75.2***	16 965	74.0***
All other race/ethnicity groups	51 905	72.5***	26 340	74.2***	21 429	73.9***	4911	75.3***
Missing race/ethnicity	44 833	68.2***	21 117	75.5***	18 573	74.9***	2544	79.3
State does not have sufficient race/ethnicity data quality	604 701	63.7***	347 141	75.6***	239 560	78.0***	107 581	70.3***
Urban/Rural residence								
Urban^^cc^	1 156 957	67.3	614 434	75.1	488 359	76.2	126 075	71.0
Rural	279 314	67.9***	151 619	79.3***	104 877	80.3***	46 742	77.1***
Eligibility Status 12 months after birth								
Parent^^^			407 980	76.3	305 977	77.8	102 003	72.0
Adult Expansion^c^			181 146	75.3***	180 769	75.3***	377	76.1*
Pregnancy			39 893	74.9***	23 924	75.7***	15 969	73.8***
Transitional Medical Assistance			46 014	78.4***	34 777	79.3***	11 237	75.8***
Child			30 881	78.5***	20 592	78.9***	10 289	77.7***
SSI			17 068	84.5***	11 072	85.2***	5996	83.3***
Other Eligibility Status			12 995	70.0***	3260	76.9	9735	67.6***
Missing or Multiple Eligibility Status			34 838	68.6***	17 428	67.7***	17 410	69.6***
Condition During Pregnancy/Birth								
No condition^^^	498 384	61.2	238 948	71.9	186 028	73.0	52 920	68.2
Preexisting chronic condition	318 860	73.2***	179 353	79.3***	140 540	80.3***	38 813	75.9***
Preterm birth	202 023	70.8***	116 803	78.9***	87 943	79.9***	28 860	76.0***
Cesarean Section	398 392	72.6***	213 193	76.7***	164 803	77.7***	48 390	73.2***
Pregnancy or delivery complication	348 182	71.3***	186 121	77.9***	144 924	78.8***	41 197	74.7***
Mental or behavioral health diagnosis	325 379	74.2***	218 781	81.9***	169 436	82.6***	49 345	79.3***

Source: 2018-2019 Transformed Medicaid Statistical Information System Analytic Files from 45 states plus DC. Sample includes enrollees with Medicaid-covered births in 2018 (see Appendix 1 for details). ^a^Race/ethnicity comparisons are limited to the 20 states with sufficient race/ethnicity data quality to identify non-Hispanic white, non-Hispanic Black, and Hispanic enrollees. Analyses of other race/ethnicity groups for states with sufficient data quality are provided in Appendix 4. ^b^4762 enrollees are excluded from the comparisons by urban/rural residence due to missing data on the Rural-Urban Commuting Area code. Adjusted comparisons are provided in Appendix 5. State Medicaid expansion status is as of January 2018. NH = non-Hispanic. SSI = Supplemental Security Income. ^c^Since we classified expansion status as of January 2018, a small number of enrollees from “non-expansion” states that implemented Medicaid expansion during the study period had an eligibility pathway of “adult expansion” at 12 months postpartum. */**/*** Estimate differs significantly from reference group (^^^) at the 0.10/0.05/0.01 level, using two-tailed tests.

Table 2 shows the shares of enrollees with at least one visit including each type of care between 61 days and 12 months postpartum, among those maintaining continuous enrollment for 12 months, by individual characteristics. In the 20 states where we were able to examine differences by race/ethnicity, Black and Hispanic enrollees were less likely to have a visit including mental/behavioral health care or a visit for other acute or chronic illness compared to white enrollees (mental/BH: 13.3%, 10.1%, and 29.4% for Black, Hispanic, and white, respectively; acute or chronic: 58.9%, 59.6%, and 65.1%), but Black and Hispanic enrollees were similarly or more likely than white enrollees to have a visit including postpartum evaluation, preventive/well care, or contraceptive management (postpartum evaluation: 3.9%, 4.1%, 3.5%; preventive/well care: 21.4%, 18.7%, 18.1%; contraceptive management: 29.5%, 32.7%, 26.4%). Adjusting for other observable characteristics, Black enrollees were 12.5 p.p. less likely and Hispanic enrollees were 11.8 p.p. less likely to have a visit that included mental/behavioral health care than white enrollees (Appendix 5). Rural enrollees were more likely than urban enrollees to have a visit that included mental/behavioral health care (25.3% vs 16.6% for rural and urban, respectively; adjusted difference, 6.6 p.p.; Appendix 5) or a visit for other acute or chronic illness (66.8% vs 60.5%; adjusted difference, 6.8 p.p.; Appendix 5) but less likely than urban enrollees to have a visit that included preventive/well care (15.9% vs 21.6%; adjusted difference, −4.7 p.p.; Appendix 5). Enrollees with a health condition or complication were more likely to have a visit that included mental/behavioral health care or a visit for other acute or chronic illness compared to those without the conditions/complications, but differences by complications/conditions in preventive/well care and contraceptive management were small. These patterns by individual characteristics were similar in expansion and non-expansion states.

**Table 2.** Share of Medicaid enrollees with a visit including postpartum evaluation, preventive/well care, contraceptive management, mental/behavioral health care, and care for other acute or chronic illness between 61 days and 12 months postpartum, by individual characteristics, among those with 12 months continuous enrollment, 2018.

	Postpartum evaluation			Well/preventive care			Contraceptive management			Mental/behavioral health care			Acute or chronic illness		
	All states in sample (%)	Expansion states (%)	Non-expansion states (%)	All states in sample (%)	Expansion states (%)	Non-expansion states (%)	All states in sample (%)	Expansion states (%)	Non-expansion states (%)	All states in sample (%)	Expansion states (%)	Non-expansion states (%)	All states in sample (%)	Expansion states (%)	Non-expansion states (%)
All	3.6	3.8	3.0	20.5	22.1	14.9	28.1	28.3	27.2	18.3	18.0	19.3	61.7	62.8	58.3
Age in years															
Less than 19^^^	3.6	3.8	3.3	21.2	23.3	17.9	40.2	40.9	39.0	16.1	16.4	15.7	62.2	62.9	61.1
19-24	3.7	3.8	3.3	19.0***	20.6***	14.0***	31.6***	32.2***	29.5***	17.0***	16.9**	17.5***	60.6***	61.6**	57.6***
25-29	3.6	3.7	2.9***	20.4***	21.9***	14.6***	27.1***	27.6***	25.2***	19.3***	19.1***	20.4***	61.3***	62.5***	57.2***
30-34	3.6	3.8	2.6***	21.6*	23.2	15.2***	23.8***	24.2***	22.2***	19.7***	19.1***	22.3***	62.7***	63.7***	58.5***
35 plus	3.7	3.9	2.5***	23.1***	24.6***	15.7***	20.0***	20.2***	18.8***	18.3***	17.4***	22.4***	64.8***	65.6***	60.9***
Race/Ethnicity^a^															
White NH^^^	3.5	3.7	2.8	18.1	18.9	13.3	26.4	26.2	27.9	29.4	28.9	31.6	65.1	65.0	65.2
Hispanic	4.1***	4.0***	5.0***	18.7***	18.8	18.3***	32.7***	32.8***	31.5***	10.1***	9.7***	13.4***	59.6***	59.5***	60.8***
Black NH	3.9***	4.0***	3.5***	21.4***	22.5***	16.7***	29.5***	28.3***	34.8***	13.3***	13.2***	13.7***	58.9***	59.5***	56.0***
All other race/ethnicity groups	3.7	4.0**	2.7	18.1	19.3	12.6	26.6	25.9	29.8***	10.3***	8.9***	16.7***	61.4***	61.2***	62.4***
Missing race/ethnicity	4.2***	4.3***	3.4	21.1***	21.6***	16.9***	25.9	25.5*	28.9	17.4***	16.0***	27.6***	62.4***	61.9***	66.0***
State does not have sufficient race/ethnicity data quality	3.4***	3.7	2.7	22.1***	25.5***	14.6***	27.2***	28.1***	25.1***	17.8***	17.7***	17.9***	61.6***	64.0***	56.2***
Urban/rural residence															
Urban^^b^	3.7	3.9	3.1	21.6	23.2	15.5	28.0	28.3	26.8	16.6	16.2	18.0	60.5	61.6	56.1
Rural	3.3***	3.5***	2.7***	15.9***	17.0***	13.5***	28.3**	28.2	28.4***	25.3***	26.4***	22.8***	66.8***	68.0***	64.0***
Eligibility status 12 months after birth															
Parent^^^	3.6	4.0	2.6	20.4	22.4	14.4	28.9	29.5	27.0	18.3	17.8	20.1	61.8	63.3	57.2
Adult expansion	3.5**	3.5***	3.7	21.7***	21.7***	11.4	26.3***	26.3***	20.7***	18.1**	18.1***	28.4***	61.3**	61.3***	63.7***
Pregnancy	5.9***	5.1***	7.1***	15.8***	18.5***	11.9***	18.1***	18.4***	17.7***	13.7***	13.9***	13.4***	66.4***	67.1***	65.3***
Transitional medical assistance	3.1***	3.3***	2.4	19.9**	21.7***	14.5	29.8***	30.2**	28.5***	19.9***	20.0***	19.8	64.0***	64.7***	61.6
Child	3.3***	3.4***	3.1***	23.9***	26.0***	19.6***	39.6***	39.3***	40.0***	17.2***	17.1**	17.5***	62.3***	62.9**	61.0***
SSI	3.4	3.6*	2.9*	22.2***	24.8***	17.3***	30.6***	30.4**	31.0***	36.4***	36.1***	37.1***	70.5***	72.0***	67.7***
Other Eligibility Status	2.5***	4.3	2.0***	16.1***	24.5***	13.3***	25.3***	26.3***	25.0***	17.0***	19.2**	16.2***	57.0***	66.3**	53.9***
Missing or multiple eligibility status	3.0***	3.2***	2.8*	19.0***	19.7***	18.4***	26.8***	24.5***	29.1***	14.8***	13.2***	16.3***	52.8***	52.2***	53.3***
Condition during pregnancy/birth															
No condition^^^	3.7	3.9	3.0	19.8	21.2	14.9	28.6	28.9	27.7	9.8	9.4	11.2	57.2	58.3	53.3
Preexisting chronic condition	3.7	3.8	3.1	21.8***	23.6***	15.3	27.3***	27.6***	26.1***	19.6***	19.4***	20.6***	67.3***	68.2***	64.0***
Preterm birth	4.1***	4.2***	3.6***	21.3***	23.2***	15.4*	29.7***	30.0***	28.9***	21.2***	21.2***	21.5***	65.5***	66.5***	62.6***
Cesarean section	3.2***	3.4***	2.5***	21.5***	23.4***	15.0	24.6***	24.9***	23.6***	20.4***	20.1***	21.4***	63.5***	64.5***	59.8***
Pregnancy or delivery complication	3.8**	4.0**	3.0	21.5***	23.3***	15.3	27.1***	27.4***	26.1***	19.2***	18.8***	20.5***	65.3***	66.2***	61.9***
Mental or behavioral health diagnosis	3.6	3.8*	3.0	19.5**	21.0	14.3***	28.7	28.9	27.9	37.7***	37.7***	37.5***	66.3***	67.2***	63.3***

Source: 2018-2019 Transformed Medicaid Statistical Information System Analytic Files from 45 states plus DC. Sample includes enrollees with Medicaid-covered births in 2018 (see Appendix 1 for details) who maintained continuous enrollment for the 12-month postpartum period. A small number (3.3%) of visits include more than one type of care. ^a^Race/ethnicity comparisons are limited to the 20 states with sufficient race/ethnicity data quality to identify non-Hispanic white, non-Hispanic Black, and Hispanic enrollees. Analyses of other race/ethnicity groups for states with sufficient data quality are provided in Appendix 4. ^b^Reference category for urban vs rural also includes 4762 enrollees with an unknown urban/rural status due to missing data on the Rural-Urban Commuting Area code. Adjusted comparisons are provided in Appendix 5. Sample size for all states is 252 375; 195 197 for expansion states, and 51 179 for non-expansion states; see Table 1 for sample sizes for each cell. State Medicaid expansion status is as of January 2018. NH = non-Hispanic. SSI is Supplemental Security Income. */**/*** Estimate differs significantly from reference group (^^^) at the 0.10/0.05/0.01 level, using two-tailed tests.

**Figure 2.**
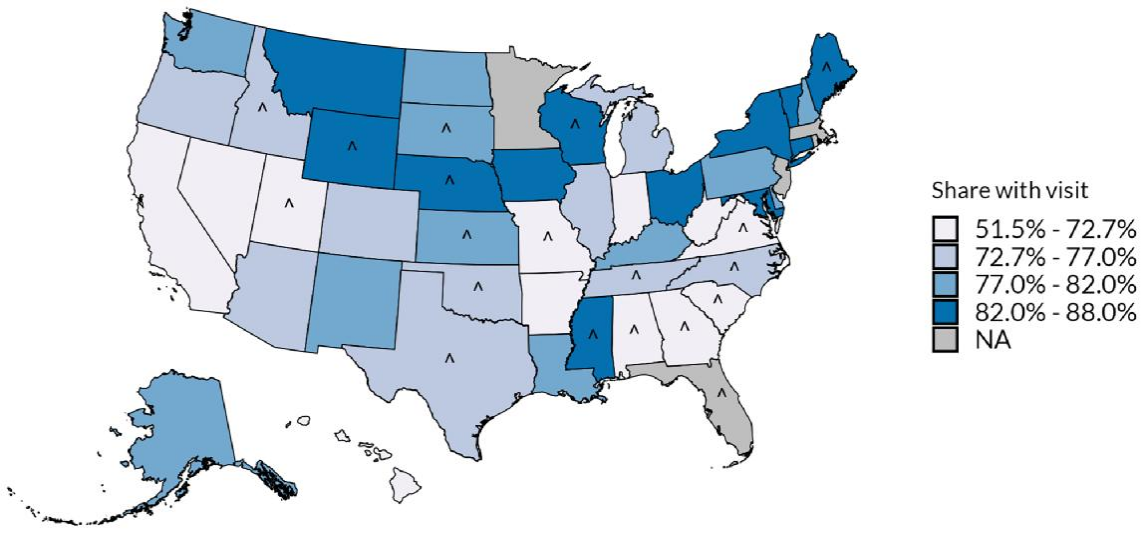
Share of Medicaid enrollees with at least one outpatient visit between 61 days and 12 months postpartum, among those with 12 months continuous enrollment, 2018 (*N* = 770 815). Source/Notes: SOURCE 2018-2019 Transformed Medicaid Statistical Information System Analytic Files from 45 states plus DC. NOTES Sample includes enrollees with Medicaid-covered births in 2018 with continuous enrollment in Medicaid through 12 months postpartum (see Appendix 1 for details). Adjusted comparisons are provided in Appendix 5. State Medicaid expansion status is as of January 2018. ^ represents non-expansion states.

Figure 2 depicts state variation in the share of enrollees with at least one visit between 61 days and 12 months postpartum among those with continuous enrollment for 12 months. Across all states, the state-level share of enrollees with a visit ranged from 51.5% to 88.0%; across expansion states, the state-level share ranged from 64.3% to 88.0%. States with the highest share included Connecticut, Vermont, Maine, Iowa, and Mississippi; in each of these states, over 85% of enrollees had a visit. States with the lowest share included Alabama, West Virginia, Arkansas, Virginia, and Georgia; in each of these states, fewer than 67% had a visit. After adjusting for other observable characteristics, differences across states were similar in magnitude (Appendix 5). For example, the adjusted difference between Connecticut and Alabama was 37.2 p.p. (compared to an unadjusted difference of 36.5 p.p.) and the adjusted difference between Connecticut and Arkansas (both expansion states) was 25.7 p.p. (compared to an unadjusted difference of 23.5 p.p.).

## Discussion

Access to affordable, high-quality outpatient health care during the postpartum period is critical to addressing the unique physical, mental, and emotional needs of people who have recently given birth. While pregnancy-related Medicaid eligibility has historically expired after 60 days postpartum, most states have recently extended Medicaid eligibility to cover up to 12 months postpartum. This study provides new evidence on Medicaid outpatient service use during the postpartum year, using near-national data on Medicaid-covered births in 2018. We find that among individuals with continuous Medicaid coverage during the postpartum year, 76.0% had at least one visit between 61 days and 12 months postpartum; 28.1% had a visit including contraceptive management; 20.5% had a visit including preventive/well care; 18.3% had a visit including mental/behavioral health care; and 61.7% had a visit including care for an acute or chronic illness. These visit rates are lower than rates among commercially insured populations identified in previous research, particularly for receipt of preventive/well care where commercially insured visit rates are estimated to be over 40%.^30^

We also examined differences by individual characteristics, which could identify where there could be inequities in access to care under the postpartum extensions. Adjusting for other observed characteristics, rural enrollees were 6.6 p.p. more likely to have a visit that included mental/behavioral health care and 6.8 p.p. more likely to have a visit for acute or chronic illness than urban enrollees, but 4.7 p.p. less likely to have a visit that included preventive/well care than urban enrollees. While we were only able to assess differences by race/ethnicity in a subset of the study states due to data quality concerns, white enrollees were 2.9 p.p. more likely than Hispanic enrollees and 3.0 p.p. more likely than Black enrollees to have a visit between 61 days and 12 months postpartum, adjusting for other observed characteristics, and these differences were driven by higher shares of white enrollees having visits that included mental/behavioral health care compared with Hispanic and Black enrollees. These differences suggest a need for further research to better understand and address the barriers that Black and Hispanic enrollees face in accessing mental/behavioral health care during the postpartum year to help ensure that Medicaid postpartum extensions improve racial equity in maternal outcomes. Finally, enrollees with chronic conditions, behavioral/mental health conditions, or pregnancy/delivery complications were more likely than those without conditions or complications to have a visit. However, some enrollees with these conditions or complications did not receive any Medicaid-covered outpatient care during the postpartum year, and we do not know from this analysis whether the visit(s) these enrollees received were adequate for addressing their healthcare needs.

This analysis also identified sizable differences across states in the share of enrollees with outpatient visits between 61 days and 12 months postpartum, ranging from just over 50% to over 85%. Much of this variability is likely driven by differences in the composition of the continuously enrolled populations across states, due in particular to the higher income eligibility thresholds for parents in expansion states than non-expansion states. However, state variation is considerable even when adjusting for individual characteristics or limiting to more “apples-to-apples” comparisons (given the similarity in their eligibility thresholds) among enrollees in states with Medicaid expansions. It will be important for future research to further decompose this variation across states to identify policy choices and delivery system characteristics in the high-performing states that could promote access to care during the postpartum year.

Our findings are consistent with smaller state-specific studies documenting the receipt of postpartum care in Medicaid. We extend the literature by focusing on the period of time 61 days to 12 months postpartum, providing estimates for a near-national population of postpartum Medicaid enrollees, and assessing the association between enrollee characteristics and visits, overall and by types of care provided.^18-20^ Given other research that has identified significant unmet healthcare needs among postpartum Medicaid populations,^31^ future work should further examine the volume and content of outpatient care, including receipt of prescription drugs as well as inpatient services used by Medicaid enrollees during the postpartum year, to assess the extent to which they are addressing the full range of health needs for different subgroups and states and should assess the association between postpartum care and longer-term health outcomes for families.

In sum, this study provides new evidence on Medicaid-covered health care use during a unique and critical period of time in people's lives and is instructive for considering the extent to which the recently implemented 12-month postpartum Medicaid extensions could help meet the health care needs of postpartum individuals and reduce inequities in maternal health outcomes. Although the population studied in this analysis does not fully reflect the population that will be affected by the postpartum extensions, our findings nonetheless suggest that the majority of postpartum Medicaid enrollees will seek outpatient visits during the postpartum year if they maintain coverage, but that there may be inequities in access to care across and within states. As 12-month postpartum Medicaid extensions are implemented across the country, effective communication of the policies by states and managed care plans, as well as policies and incentives to ensure adequate delivery system capacity and availability for postpartum Medicaid enrollees, will be paramount to ensure enrollees are made aware of their extended coverage and how it can facilitate their access to affordable health care that meets their needs.^32,33^

## Supplementary Material

qxaf019_Supplementary_Data

## References

[1] Gunja MZ, Gumas ED, Masitha R, Zephyrin LC. Insights into the U.S. Maternal Mortality Crisis: An International Comparison. The Commonwealth Fund; 2024.

[2] CDC . Pregnancy-related deaths: data from maternal mortality review committees in 36 U.S. States, 2017–2019. Maternal Mortality Prevention. May 30, 2024. Accessed June 6, 2024. https://www.cdc.gov/maternal-mortality/php/data-research/mmrc-2017-2019.html

[3] CDC . Working together to reduce black maternal mortality. Women's Health. June 16, 2024. Accessed September 3, 2024. https://www.cdc.gov/womens-health/features/maternal-mortality.html

[4] Rep. Yarmuth JA [D K 3 . Text—H.R.1319—117^th^ Congress (2021-2022): American Rescue Plan Act of 2021. March 11, 2021. Accessed June 6, 2024. https://www.congress.gov/bill/117th-congress/house-bill/1319/text

[5] Centers for Medicare and Medicaid Services. SHO# 21-007 RE: improving maternal health and extending postpartum coverage in Medicaid and the Children's Health Insurance Program (CHIP). Published online December 7, 2021. Accessed November 1, 2024. https://www.medicaid.gov/federal-policy-guidance/downloads/sho21007.pdf.

[6] Valenzuela CP, Osterman MJK. Characteristics of Mothers by Source of Payment for the Delivery: United States, 2021. National Center for Health Statistics; 2023. 10.15620/cdc:127266 PMC13659882 37256286

[7] Published: Medicaid Postpartum Coverage Extension Tracker . KFF. May 10, 2024. Accessed June 6, 2024. https://www.kff.org/medicaid/issue-brief/medicaid-postpartum-coverage-extension-tracker/

[8] Haley JM, Johnston EM, Hill I, Kenney GM, Thomas TW. The Public Health Insurance Landscape for Pregnant and Postpartum Women: State and Federal Policies in 2020. Urban Institute; 2021.

[9] Johnston EM, McMorrow S, Alvarez Caraveo C, Dubay L. Post-ACA L, more than one-third of women with prenatal Medicaid remained uninsured before or after pregnancy. Health Aff (Millwood). 2021;40(4):571–578. 10.1377/hlthaff.2020.01678 33819081

[10] Brooks T, Wagnerman K, Artiga S, Published EC. Medicaid and CHIP eligibility, enrollment, renewal, and cost sharing policies as of january 2018: findings from a 50-state survey. KFF. March 21, 2018. Accessed October 9, 2024. https://www.kff.org/medicaid/report/medicaid-and-chip-eligibility-enrollment-renewal-and-cost-sharing-policies-as-of-january-2018-findings-from-a-50-state-survey/

[11] McMorrow S, Haley JM, Johnston EM. The American Rescue Plan Contains an Evidence-Based Policy Win for New Mothers. Urban Institute; 2021.

[12] McMorrow S, Dubay L, Kenney GM, Johnston EM, Caraveo CA. Uninsured New Mothers’ Health and Health Care Challenges Highlight the Benefits of Increasing Postpartum Medicaid Coverage. Urban Institute; 2020.

[13] Optimizing Postpartum Care . Accessed July 25, 2024. https://www.acog.org/clinical/clinical-guidance/committee-opinion/articles/2018/05/optimizing-postpartum-care

[14] Attanasio LB, Ranchoff BL, Cooper MI, Geissler KH. Postpartum visit attendance in the United States: a systematic review. Womens Health Issues. 2022;32(4):369–375. 10.1016/j.whi.2022.02.002 35304034 PMC9283204

[15] Taylor YJ, Liu T-L, Howell EA. Insurance differences in preventive care use and adverse birth outcomes among pregnant women in a Medicaid nonexpansion state: a retrospective cohort study. J Womens Health. 2020;29(1):29–37. 10.1089/jwh.2019.7658 PMC6983742 31397625

[16] Wilcox A, Levi EE, Garrett JM. Predictors of non-attendance to the postpartum follow-up visit. Matern Child Health J. 2016;20(S1):22–27. 10.1007/s10995-016-2184-9 27562797

[17] Centers for Medicare and Medicaid Services . Quality of care for adults in Medicaid: findings from the 2020 adult core set chart pack. Accessed November 1, 2024. https://www.medicaid.gov/medicaid/quality-of-care/downloads/performance-measurement/2021-adult-chart-pack.pdf.

[18] Gordon SH, Lee S, Steenland MW, Deen N, Feinberg E. Extended postpartum medicaid in Colorado associated with increased treatment for perinatal mood and anxiety disorders. Health Aff (Millwood). 2024;43(4):523–531. 10.1377/hlthaff.2023.01441 38560800 PMC12038864

[19] Wang X, Pengetnze YM, Eckert E, Keever G, Chowdhry V. Extending postpartum medicaid beyond 60 days improves care access and uncovers unmet needs in a Texas Medicaid Health Maintenance Organization. Front Public Health. 2022;10:841832. 10.3389/fpubh.2022.841832 35592081 PMC9110670

[20] Gordon SH, Sommers BD, Wilson IB, Trivedi AN. Effects of medicaid expansion on postpartum coverage and outpatient utilization. Health Aff (Millwood). 2020;39(1):77–84. 10.1377/hlthaff.2019.00547 31905073 PMC7926836

[21] Auty SG, Daw JR, Admon LK, Gordon SH. Comparing approaches to identify live births using the Transformed Medicaid Statistical Information System. Health Serv Res. 2024;59(1):e14233. 10.1111/1475-6773.14233 37771156 PMC10771902

[22] Admon LK, Auty SG, Daw JR, et al State variation in severe maternal morbidity among individuals with Medicaid insurance. Obstet Gynecol. 2022;141(5):877–885. 10.1097/AOG.0000000000005144 PMC10281794 37023459

[23] Welcome | DQ Atlas . Accessed March 2, 2022. https://www.medicaid.gov/dq-atlas/welcome

[24] SSA O . State plans for medical assistance. Accessed August 20, 2024. https://www.ssa.gov/OP_Home/ssact/title19/1902.htm

[25] USDA ERS—Rural-Urban Commuting Area Codes . Accessed March 14, 2022. https://www.ers.usda.gov/data-products/rural-urban-commuting-area-codes/

[26] Clinical Episode Payment Models Chapter 4: Maternity Care . Health Care Payment Learning & Action Network. 2016. Accessed November 1, 2024. https://hcp-lan.org/workproducts/maternity-whitepaper-final.pdf.

[27] Centers for Medicare and Medicaid Services . Lessons learned about payment strategies to improve postpartum care in medicaid and CHIP. 2019. Accessed November 1, 2024. https://www.medicaid.gov/medicaid/quality-of-care/downloads/postpartum-payment-strategies.pdf.

[28] Ranji U, Gomez I, Salganicoff A, Rosenzweig C, Kellenberg R, Published KG. Medicaid coverage of pregnancy-related services: findings from a 2021 State Survey—Report—9936. KFF. May 19, 2022. Accessed June 10, 2024. https://www.kff.org/report-section/medicaid-coverage-of-pregnancy-related-services-findings-from-a-2021-state-survey-report/

[29] Daw JR, Auty SG, Admon LK, Gordon SH. Using modernized medicaid data to advance evidence-based improvements in maternal health. Am J Public Health. 2023;113(7):805–810. 10.2105/AJPH.2023.307287 37141557 PMC10262233

[30] Steenland MW, Kozhimannil KB, Werner EF, Daw JR. Health care use by commercially insured postpartum and nonpostpartum women in the United States. Obstet Gynecol. 2021;137(5):782. 10.1097/AOG.0000000000004359 33831924 PMC8058261

[31] Daw JR, Underhill K, Liu C, Allen HL. The health and social needs of Medicaid beneficiaries in the postpartum year: evidence from a multistate survey. Health Aff (Millwood). 2023;42(11):1575–1585. 10.1377/hlthaff.2023.00541 37931190

[32] Johnston EM, Haley JM, Long J, Kenney GM. New Mothers’ Coverage Improved During the Public Health Emergency. Urban Institute; 2023.

[33] Allen EH, Haley JM, Verdeflor A, Dudley K. Improving Maternal Health and Well-Being Through Medicaid/CHIP Postpartum Coverage Extensions. Urban Institute; 2024.

