# Correction to: Medicaid-covered health care visits during the postpartum year: Variation by enrollee characteristics and state

**DOI:** 10.1093/haschl/qxaf055

**Published:** 2025-03-21

This is a **correction** to:

Laura Barrie Smith, Claire O’Brien, Keqin Wei, Timothy A Waidmann, Genevieve M Kenney, Medicaid-covered health care visits during the postpartum year: Variation by enrollee characteristics and state, *Health Affairs Scholar*, Volume 3, Issue 2, February 2025, qxaf019, https://doi.org/10.1093/haschl/qxaf019

In the originally published version of this manuscript, the “Acknowledgement Section” was erroneously left off.

This error has been corrected and the following funding statement has been included:

This work was supported by funding from the Robert Wood Johnson Foundation

