# Supplementary material for: Medicaid-covered health care visits during the postpartum year: Variation by enrollee characteristics and state
Source: Health Aff Sch. 2025 Jan 30;3(2):qxaf019. doi: 10.1093/haschl/qxaf019 (PMC11823106; doi:10.1093/haschl/qxaf019)
Supplement: qxaf019_Supplementary_Data [file qxaf019_supplementary_data.zip › Postpartum visit APPENDIX revised 01.27.25 V2.docx]

Table of Contents

[Appendix 1: Identifying study population of Medicaid enrollees with live births, 2018 2](#_Toc188880321)

[Appendix 2: Enrollee characteristics by continuous enrollment status during 12 months postpartum 3](#_Toc188880322)

[Appendix 3: Definitions of outpatient visits, types of care provided, and health conditions 4](#_Toc188880323)

[Appendix 4: Share of Medicaid enrollees with visits within 60 days postpartum and between 61 days and 12 months postpartum by race/ethnicity, 2018 deliveries 7](#_Toc188880324)

[Appendix 5: Regression output for the association between enrollee characteristics and postpartum visits between 61 days and 12 months postpartum, 2018 9](#_Toc188880325)

[Appendix 6: Share of Medicaid enrollees with visits within 60 days and between 61 days and 12 months postpartum by individual characteristics, limited to expansion-eligible enrollees in ACA expansion states, 2018 14](#_Toc188880326)

[Appendix 7: Share of postpartum enrollees with postpartum evaluation visits within 60 days postpartum and between 61 days and 12 months postpartum, by state frequency of bundled payment codes for prenatal care, 2018 15](#_Toc188880327)

[Appendix 8: Share of Medicaid enrollees with visits within 60 days postpartum and between 61 days and 12 months postpartum, by individual characteristics, September-December 2018 births 16](#_Toc188880328)

[Appendix 9: Share of Medicaid enrollees with at least one outpatient visit within the full 12-month postpartum period, among those with continuous enrollment for 12 months, 2018 deliveries (N= 770,815) 18](#_Toc188880329)

[Appendix 10: Share of Medicaid enrollees with at least one outpatient visit within 60 days postpartum, among those with continuous enrollment for 12 months, 2018 (N= 770,815) 19](#_Toc188880330)

[Appendix 11: Share of Medicaid enrollees with visits during the full 12-month postpartum period by individual characteristics, 2018 20](#_Toc188880331)

## Appendix 1: Identifying study population of Medicaid enrollees with live births, 2018

| Exclusion | Number of enrollees remaining | Number of enrollees excluded from last step | Share of enrollees excluded from last step | Share of the starting population |
| --- | --- | --- | --- | --- |
| All enrollees with a Medicaid-covered live-birth delivery occurring in 2018 in the 46 study states^+^ | 1,534,857 | 23,026,273 | 93.8% | 100% |
| Exclude enrollees in more than one state during 60-day postpartum period | 1,491,084 | 43,773 | 2.9% | 97.1% |
| Exclude enrollees dually eligible for Medicare during 60-day postpartum period | 1,480,555 | 10,529 | 0.7% | 96.5% |
| Exclude enrollees with zero days of Medicaid enrollment during 60-day postpartum period | 1,453,767 | 37,317 | 2.5% | 94.7% |
| For the sample of those with 12 months continuous enrollment: | | | | |
| Exclude enrollees with less than 360 days Medicaid enrollment during 12-month postpartum period | 867,761 | 612,794 | 41.4% | 56.5% |
| Exclude enrollees dually eligible for Medicare during 12-month postpartum period | 867,027 | 734 | 0.1% | 56.5% |
| Exclude enrollees with restricted Medicaid benefits during 12-month postpartum period | 770,815 | 96,212 | 11.1% | 50.2% |

SOURCE: 2018-2019 Transformed Medicaid Statistical Information System Analytic Files from 45 states plus the District of Columbia.
NOTES: TAF is Transformed Medicaid Statistical Information System Analytic Files. CHIP is Children's Health Insurance Program. State specific sample creation tables are available upon request. ^+^Florida, Minnesota, Massachusetts, New Jersey, and Rhode Island were excluded due to “high concern” about the volume of their outpatient claims in the 2018 TAF, according to <https://www.medicaid.gov/dq-atlas/landing/topics/single/map?topic=g15m47&tafVersionId=18>. For comparisons by race and ethnicity, the following 26 states were excluded due to concerns about the quality of their race and ethnicity variable (see Appendix 4 for details): Alabama, Alaska, Arizona, Arkansas, Colorado, Connecticut, District of Columbia, Georgia, Hawaii, Iowa, Idaho, Kansas, Louisiana, Michigan, Missouri, Mississippi, Montana, New York, Oregon, South Carolina, Tennessee, Utah, Virginia, Vermont, West Virginia, and Wyoming.

## Appendix 2: Enrollee characteristics by continuous enrollment status during 12 months postpartum

|  | All enrollees with Medicaid-covered births in 2018 | | Enrolled for less than 12 months postpartum | | Continuously enrolled for 12 months postpartum | | |
| --- | --- | --- | --- | --- | --- | --- | --- |
|  | N | Share | N | Share | N | Share | |
| All | 1,453,767 | 100.0% | 682,952 | 100.0% | 770,815 | 100.0% |  |
| Age in years |  |  |  |  |  |  |  |
| Less than 19 | 72,084 | 5.0% | 24,250 | 3.6% | 47,834 | 6.2% | *** |
| 19-24 | 492,196 | 33.9% | 229,291 | 33.6% | 262,905 | 34.1% | *** |
| 25-29 | 447,825 | 30.8% | 210,819 | 30.9% | 237,006 | 30.7% |  |
| 30-34 | 278,484 | 19.2% | 135,736 | 19.9% | 142,748 | 18.5% | *** |
| 35 plus | 163,178 | 11.2% | 82,856 | 12.1% | 80,322 | 10.4% | *** |
| Race/Ethnicity^+^ |  |  |  |  |  |  |  |
| White NH | 286,441 | 19.7% | 119,710 | 17.5% | 166,731 | 21.6% | *** |
| Hispanic | 312,545 | 21.5% | 192,136 | 28.1% | 120,409 | 15.6% | *** |
| Black NH | 153,342 | 10.5% | 64,265 | 9.4% | 89,077 | 11.6% | *** |
| All other race/ethnicity groups | 51,905 | 3.6% | 25,565 | 3.7% | 26,340 | 3.4% | *** |
| Missing race/ethnicity | 44,833 | 3.1% | 23,716 | 3.5% | 21,117 | 2.7% | *** |
| State does not have sufficient race/ethnicity data quality | 604,701 | 41.6% | 257,560 | 37.7% | 347,141 | 45.0% | *** |
| Urban/Rural residence |  |  |  |  |  |  |  |
| Urban | 1,156,957 | 79.6% | 542,523 | 79.4% | 614,434 | 79.7% | *** |
| Rural | 279,314 | 19.2% | 127,695 | 18.7% | 151,619 | 19.7% | *** |
| Condition During Pregnancy/Birth |  |  |  |  |  |  |  |
| No condition | 498,384 | 34.3% | 259,436 | 38.0% | 238,948 | 31.0% | *** |
| Preexisting chronic condition | 318,860 | 21.9% | 139,507 | 20.4% | 179,353 | 23.3% | *** |
| Preterm birth | 202,023 | 13.9% | 85,220 | 12.5% | 116,803 | 15.2% | *** |
| Cesarean Section | 398,392 | 27.4% | 185,199 | 27.1% | 213,193 | 27.7% | *** |
| Pregnancy or delivery complication | 348,182 | 24.0% | 162,061 | 23.7% | 186,121 | 24.1% | *** |
| Mental or behavioral health diagnosis | 325,379 | 22.4% | 106,598 | 15.6% | 218,781 | 28.4% | *** |
| State ACA Medicaid expansion status |  |  |  |  |  |  |  |
| Expansion | 861,757 | 59.3% | 263,958 | 38.6% | 597,799 | 77.6% | *** |
| Non-expansion | 592,010 | 40.7% | 418,994 | 61.4% | 173,016 | 22.4% | *** |

Source: 2018-2019 Transformed Medicaid Statistical Information System Analytic Files from 45 states plus DC.

Notes: Sample includes enrollees with Medicaid-covered births in 2018 (see Appendix 1 for details). ^+^Race/ethnicity comparisons are limited to the 20 states with sufficient race/ethnicity data quality (see Appendix 4). State Medicaid expansion status is as of January 2018. NH= non-Hispanic. SSI= Supplemental Security Income. */**/*** Estimate differs significantly between groups at the 0.10/0.05/0.01 level, using two-tailed tests.

## Appendix 3: Definitions of outpatient visits, types of care provided, and health conditions

Definition of outpatient visits, overall

We used the TAF Other Services (OT) file to identify all outpatient visits, which excludes services provided in an inpatient, emergency department, and urgent care setting. For the primary outcome of “any outpatient visit,” we defined the measure to broadly capture outpatient visits to ensure our findings on overall visit rates were not driven by differences in the specific diagnosis and/or procedure codes providers used to bill for services provided. This broad measure included claims with any of the following codes:

CPT codes: 99201 99202 99203 99204 99205 99211 99212 99213 99214 99215 99241 99242 99243 99244 99245 99341 99342 99343 99344 99345 99347 99348 99349 99350 99381 99382 99383 99384 99385 99386 99387 99391 99392 99393 99394 99395 99396 99397 99401 99402 99403 99404 99411 99412 99429 92002 92004 92012 92014 99304 99305 99306 99307 99308 99309 99310 99315 99316 99318 99324 99325 99326 99327 99328 99334 99335 99336 99337 98966 98967 98968 99441 99442 99443 98969 99444 99483; HCPCS code: G0402 G0438 G0439 G0463 T1015 S0620 S0621; Procedure code modifiers: 95 and GT; Place of service code: 02; ICD-10 code: Z0000 Z0001 Z00121 Z00129 Z003 Z005 Z008 Z020 Z021 Z022 Z023 Z024 Z025 Z026 Z0271 Z0279 Z0281 Z0282 Z0283 Z0289 Z029 Z761 Z762; Revenue code: 0510 0511 0512 0513 0514 0515 0516 0517 0519 0520 0521 0522 0523 0526 0527 0528 0529 0982 0983 0524 0525

Defining types of care provided during outpatient visits

We classified outpatient visits into the following non-mutually-exclusive categories of care provided based on diagnosis and/or procedure codes identified on a visit: postpartum evaluation, preventive/well care, contraceptive management, mental/behavioral health care, and care for another chronic or acute illness. These categories are not mutually exclusive because an individual claim may include multiple diagnosis and/or procedure codes belonging to different types of care, or an enrollee may have multiple claims on the same day belonging to different types of care. However, if a given visit includes postpartum evaluation, preventive/well care, contraceptive management, and/or mental health care, it will not also get classified as “care for another chronic or acute illness.” For example, if a claim included a diagnosis code for a postpartum evaluation (Z39), a diagnosis code contraceptive management (Z30), and a procedure code for an evaluation and monitoring office visit (99213), that visit would be classified as including postpartum evaluation and contraceptive management, but would not also be classified as including care for another chronic or acute illness. Across all visits for all enrollees in the study population during the postpartum year, approximately 3.3% were classified into more than one category; over half of these fell into “postpartum evaluation” and “contraceptive management.” See Table A3.1 for details.

- Postpartum evaluation: ICD-10 code: Z39
- Preventive/well care: Procedure code: 99384-99387 99394-99397 99381 99382 99383 99391 99392 99393 99401 99402 99403 99404 99411 99412 99429; Diagnosis code: Z00.00, Z00.01, Z00.121, Z00.129
- Contraceptive management: ICD-10 code: Z30
- Mental /behavioral health care: Procedure codes: 90804 90805 90806 90807 90808 90809 90810 90811 90812 90813 90814 90815 90816 90817 90818 90819 90821 90822 90823 90824 90826 90827 90828 90829 90832 90833 90834 90836 90837 90838 90839 90840 90845 90846 90847 90849 90853 90857 90862 90875 90876 90820 90841 90842 90843 90844 90855 (see Gordon SH, Lee S, Steenland MW, Deen N, Feinberg E. Extended Postpartum Medicaid In Colorado Associated With Increased Treatment For Perinatal Mood And Anxiety Disorders. Health Aff (Millwood). 2024;43(4):523-531. doi:10.1377/hlthaff.2023.01441); or procedure codes 99201-99205 or 99211-99215 AND ICD-10 codes including: F01-F09 F10-F19 F20-F29 F30-F39 F40-F48 F50-F59 F60-F69 F70-F79 F80-F89 F99 O99.34
- Other acute or chronic illness: all other codes not captured in the previous categories. Note: the majority of these visits had procedure codes 99210-99215 or 99201-99205 (evaluation and monitoring office visits).

*Appendix Table A3.1: Share of visits by combination of types of care*

| **Type(s) of care provided** | **Share of all visits** |
| --- | --- |
| Other acute or chronic illness only | 48.7% |
| Mental/behavioral health care only | 19.8% |
| Postpartum evaluation only | 11.2% |
| Contraceptive management only | 10.7% |
| Preventive/well care only | 6.3% |
| Postpartum & Contraceptive | 1.8% |
| Preventive/well & Contraceptive | 0.6% |
| Postpartum & Preventive/well | 0.4% |
| Contraceptive & Mental/behavioral health care | 0.3% |
| Postpartum & mental/behavioral health care | 0.2% |
| Preventive/well & mental/behavioral health | 0.2% |

Defining health conditions and pregnancy/delivery complications

For each condition in Table A3.2 below, we considered diagnosis and/or procedure codes on all inpatient (IP) and other services (OT) claims occurring January 1, 2018 through 7 days following the date of delivery. An enrollee was flagged for the condition if they had one or more instance of the relevant procedure/diagnosis codes during this time. see Appendix 8 for sensitivity analysis excluding deliveries early in the year for which we had limited claims history.

*Appendix Table A3.2: Diagnosis and procedure codes for health conditions and pregnancy/delivery complications*

| **Condition type** | **Codes** |
| --- | --- |
| Pre-existing chronic conditions prior to pregnancy: hypertension, diabetes, obesity | Diagnosis codes O10, O24.0, O24.1, O24.3, O99.21 |
| Preterm birth | Diagnosis codes O60 |
| Cesarean section | Diagnosis codes O82, O7582; procedure codes 59510, 59514, 59515, 59618, 59620, 59622 |
| Other complications of pregnancy and delivery: gestational hypertension, gestational diabetes, eclampsia, HELLP syndrome, multiple births, blood transfusion, 3^rd^ or 4^th^ degree perineal laceration, ruptured uterus, hysterectomy | Diagnosis codes O13, O24.4, O24.8, O24.9, O15, O14, Z372, Z373, Z374, Z3750, Z3751, Z3752, Z3753, Z3754, Z3759, Z3760, Z3761, Z3762, Z3763, Z3764, Z3769, Z377, O70.2, O70.3, O71.0, O71.1;  Procedure codes 30230H0, 30230K0, 30230L0, 30230M0, 30230N0, 30230P0, 30230R0, 30230T0, 30230H1, 30230K1, 30230L1, 30230M1, 30230N1, 30230P1, 30230R1, 30230T1, 30233H0, 30233K0, 30233L0, 30233M0, 30233N0, 30233P0, 30233R0, 30233T0, 30233H1, 30233K1, 30233L1, 30233M1, 30233N1, 30233P1, 30233R1, 30233T1, 30240H0, 30240K0, 30240L0, 30240M0, 30240N0, 30240P0, 30240R0, 30240T0, 30240H1, 30240K1, 30240L1, 30240M1, 30240N1, 30240P1, 30240R1, 30240T1, 30243H0, 30243K0, 30243L0, 30243M0, 30243N0, 30243P0, 30243R0, 30243T0, 30243H1, 30243K1, 30243L1, 30243M1, 30243N1, 30243P1, 30243R1, 30243T158150-58294 |
| Mental and behavioral health conditions diagnosed during pregnancy: substance use disorder, anxiety/depression/mood disorders, other | Diagnosis codes F01-F09, F10-F19, F20-F29, F30-F39, F40-F48, F50-F59, F60-F69, F70-F79, F80-F89, F99, O99.34 |

## Appendix 4: Share of Medicaid enrollees with visits within 60 days postpartum and between 61 days and 12 months postpartum by race/ethnicity, 2018 deliveries

|  | Births with 60 days continuous enrollment postpartum (i.e., all births in sample) | | | Births with 12 months continuous eligibility postpartum | | | | | | | | |
| --- | --- | --- | --- | --- | --- | --- | --- | --- | --- | --- | --- | --- |
|  | Visit within 60 days | | | Visit between 61 days and 12 months | | | | | | | | |
|  | All states in sample | | | All states in sample | | | Expansion states | | | Non-expansion states | | |
|  | N | Share (%) | | N | Share (%) | | N | Share (%) | | N | Share (%) | |
| All enrollees (N= 46 states) | 1,453,767 | 67.4 |  | 770,815 | 76.0 |  | 597,799 | 76.9 |  | 173,016 | 72.6 |  |
| States with sufficient data quality to assess White NH, Hispanic, and Black NH (N= 20 states) | | | | | | | | | | | | |
| All enrollees in sub-sample (N=20 states) | 849,066 | 70.0 |  | 423,674 | 76.3 |  | 358,239 | 76.2 |  | 65,435 | 76.5 |  |
| White NH^ | 286,441 | 73.4 |  | 166,731 | 79.1 |  | 140,459 | 79.1 |  | 26,272 | 79.1 |  |
| Hispanic | 312,545 | 66.2 | *** | 120,409 | 73.9 | *** | 105,666 | 73.7 | *** | 14,743 | 74.8 | *** |
| Black NH | 153,342 | 71.3 | *** | 89,077 | 74.9 | *** | 72,112 | 75.2 | *** | 16,965 | 74.0 | *** |
| All other race/ethnicity groups | 51,905 | 72.5 | *** | 26,340 | 74.2 | *** | 21,429 | 73.9 | *** | 4,911 | 75.3 | *** |
| Missing race/ethnicity | 44,833 | 68.2 | *** | 21,117 | 75.5 | *** | 18,573 | 74.9 | *** | 2,544 | 79.3 |  |
| States with sufficient data quality to assess White NH, Hispanic, Black NH, and Asian (N= 14 states) | | | | | | | | | | | | |
| All enrollees in sub-sample (N=14 states)^a^ | 738,481 | 69.8 |  | 350,497 | 75.6 |  | 288,668 | 75.5 |  | 61,829 | 76.1 |  |
| By race/ethnicity: |  |  |  |  |  |  |  |  |  |  |  |  |
| Asian NH^ | 27,284 | 76.4 |  | 13,874 | 72.5 |  | 12,744 | 72.8 |  | 1,130 | 68.2 |  |
| White NH | 222,447 | 73.2 | *** | 123,227 | 78.5 | *** | 99,433 | 78.5 | *** | 23,794 | 78.5 | *** |
| Hispanic | 305,186 | 66.5 | *** | 117,316 | 73.8 | *** | 102,649 | 73.7 | ** | 14,667 | 74.8 | *** |
| Black NH | 126,491 | 70.7 | *** | 68,950 | 74.4 | *** | 52,156 | 74.5 | *** | 16,794 | 73.8 | *** |
| All other race/ethnicity groups | 19,350 | 67.6 | *** | 9,538 | 76.0 | *** | 6,535 | 75.5 | *** | 3,003 | 76.9 | *** |
| Missing race/ethnicity | 37,723 | 70.4 | *** | 17,592 | 74.9 | *** | 15,151 | 74.3 | *** | 2,441 | 79.1 | *** |
| States with sufficient data quality to assess White NH, Hispanic, Black NH, and AIAN (N= 10 states) | | | | | | | | | | | | |
| All enrollees in sub-sample (N=10 states)^b^ | 535,036 | 70.1 |  | 227,154 | 74.5 |  | 169,149 | 73.8 |  | 58,005 | 76.4 |  |
| By race/ethnicity: |  |  |  |  |  |  |  |  |  |  |  |  |
| AIAN NH^ | 8,940 | 70.2 |  | 5,944 | 79.2 |  | 4,383 | 78.9 |  | 1,561 | 79.8 |  |
| White NH | 133,971 | 73.6 | *** | 63,002 | 76.8 | *** | 41,304 | 75.7 | *** | 21,698 | 79.0 |  |
| Hispanic | 264,101 | 67.9 | *** | 100,957 | 73.5 | *** | 86,857 | 73.3 | *** | 14,100 | 74.9 | *** |
| Black NH | 75,767 | 69.6 |  | 32,568 | 73.2 | *** | 16,564 | 72.6 | *** | 16,004 | 73.9 | *** |
| All other race/ethnicity groups | 28,420 | 75.3 | *** | 14,483 | 71.8 | *** | 12,051 | 71.6 | *** | 2,432 | 72.5 | *** |
| Missing race/ethnicity | 23,837 | 70.8 |  | 10,200 | 74.8 | *** | 7,990 | 73.4 | *** | 2,210 | 79.9 |  |
| States with sufficient data quality to assess White NH, Hispanic, Black NH, and Hawaiian/Pacific Islander (N= 4 states) | | | | | | | | | | | | |
| All enrollees in sub-sample (N=4 states)^c^ | 413,521 | 68.7 |  | 160,063 | 72.7 |  | 135,594 | 72.5 |  | 24,469 | 74.0 |  |
| By race/ethnicity: |  |  |  |  |  |  |  |  |  |  |  |  |
| Hawaiian/PI NH^ | 1,775 | 61.2 |  | 1,050 | 64.3 |  | 1,014 | 64.6 |  | 36 | 55.6 |  |
| White NH | 84,343 | 71.0 | *** | 33,532 | 73.7 | *** | 27,093 | 73.6 | *** | 6,439 | 73.8 | ** |
| Hispanic | 233,182 | 68.0 | *** | 86,659 | 72.6 | *** | 75,639 | 72.3 | *** | 11,020 | 74.7 | *** |
| Black NH | 49,642 | 67.0 | *** | 18,591 | 71.5 | *** | 14,347 | 71.3 | *** | 4,244 | 71.9 | ** |
| All other race/ethnicity groups | 24,552 | 71.5 | *** | 11,499 | 72.4 | *** | 10,271 | 72.3 | *** | 1,228 | 73.5 | ** |
| Missing race/ethnicity | 20,027 | 68.8 | *** | 8,732 | 73.7 | *** | 7,230 | 72.9 | *** | 1,502 | 77.2 | *** |

Source: 2018-2019 Transformed Medicaid Statistical Information System Analytic Files from 45 states plus DC.

Notes: Sample includes enrollees with Medicaid-covered births in 2018 (see Appendix 1 for details). We applied the following criteria to identify states with sufficient data quality for each race/ethnicity category: 1) less than 20% of deliveries in the TAF sample had a missing value for race/ethnicity; 2) for each race/ethnicity category, there were at least 100 deliveries in the TAF data and at least 100 births in the CDC Natality data; and 3) the share of deliveries for each race/ethnicity category in the TAF data varied by no more than 10 percentage points and no more than 50 percent from the share of births in the CDC Natality data. ^a^States with sufficient race/ethnicity data quality include California, Delaware, Illinois, Kentucky, Maryland, North Carolina, Nebraska, New Mexico, Nevada, Oklahoma, Pennsylvania, Texas, Washington, and Wisconsin. ^b^ States with sufficient race/ethnicity data quality include California, North Carolina, North Dakota, Nebraska, New Mexico, Nevada, South Dakota, Texas, Washington, and Wisconsin. ^c^ States with sufficient race/ethnicity data quality include California, Nevada, Oklahoma, and Texas. State Medicaid expansion status is as of January 2018. AIAN= American Indian Alaskan Native. NH= non-Hispanic. */**/*** Estimate differs significantly from reference group (^) at the 0.10/0.05/0.01 level, using two-tailed tests.

## Appendix 5: Regression output for the association between enrollee characteristics and postpartum visits between 61 days and 12 months postpartum, 2018

PANEL A: Enrollees with continuous 12-months enrollment in all study states (N= 770,815)

|  | Any visit | | Postpartum evaluation | | Preventive/well | | Contraceptive management | | Mental/behavioral health care | | Acute or chronic illness | |
| --- | --- | --- | --- | --- | --- | --- | --- | --- | --- | --- | --- | --- |
|  | Coefficient | P-value | Coefficient | P-value | Coefficient | P-value | Coefficient | P-value | Coefficient | P-value | Coefficient | P-value |
| Age in years (ref= less than 19) |  |  |  |  |  |  |  |  |  |  |  |  |
| 19 to 24 | -0.042 | 0.000 | -0.002 | 0.056 | -0.013 | 0.000 | -0.076 | 0.000 | 0.010 | 0.000 | -0.029 | 0.000 |
| 25 to 29 | -0.045 | 0.000 | -0.003 | 0.007 | -0.002 | 0.314 | -0.120 | 0.000 | 0.025 | 0.000 | -0.026 | 0.000 |
| 30 to 34 | -0.046 | 0.000 | -0.003 | 0.019 | 0.007 | 0.007 | -0.152 | 0.000 | 0.026 | 0.000 | -0.017 | 0.000 |
| 35+ | -0.044 | 0.000 | -0.002 | 0.110 | 0.017 | 0.000 | -0.190 | 0.000 | 0.017 | 0.000 | 0.001 | 0.763 |
| Rurality  (ref= urban^+^) |  |  |  |  |  |  |  |  |  |  |  |  |
| Rural residence | 0.042 | 0.000 | -0.003 | 0.000 | -0.047 | 0.000 | -0.002 | 0.072 | 0.066 | 0.000 | 0.068 | 0.000 |
| Eligibility status 12 months after birth  (ref= low-income parent) |  |  |  |  |  |  |  |  |  |  |  |  |
| Expansion adult | -0.021 | 0.000 | -0.003 | 0.000 | -0.003 | 0.003 | -0.029 | 0.000 | 0.004 | 0.000 | -0.017 | 0.000 |
| Pregnancy | -0.003 | 0.133 | 0.024 | 0.000 | -0.033 | 0.000 | -0.115 | 0.000 | -0.031 | 0.000 | 0.059 | 0.000 |
| Transitional Medical Assistance | 0.020 | 0.000 | -0.005 | 0.000 | -0.003 | 0.083 | 0.007 | 0.001 | 0.015 | 0.000 | 0.021 | 0.000 |
| Child | -0.001 | 0.713 | -0.005 | 0.000 | 0.047 | 0.000 | 0.020 | 0.000 | 0.000 | 0.926 | 0.001 | 0.706 |
| SSI | 0.056 | 0.000 | -0.002 | 0.291 | 0.026 | 0.000 | 0.016 | 0.000 | 0.097 | 0.000 | 0.064 | 0.000 |
| Other | -0.041 | 0.000 | -0.006 | 0.000 | -0.010 | 0.005 | -0.022 | 0.000 | -0.021 | 0.000 | -0.024 | 0.000 |
| Missing | -0.065 | 0.000 | -0.004 | 0.000 | 0.006 | 0.007 | -0.033 | 0.000 | -0.024 | 0.000 | -0.073 | 0.000 |
| Preexisting chronic condition | 0.039 | 0.000 | 0.001 | 0.145 | 0.011 | 0.000 | 0.007 | 0.000 | 0.004 | 0.000 | 0.062 | 0.000 |
| Preterm birth | 0.028 | 0.000 | 0.005 | 0.000 | 0.012 | 0.000 | 0.017 | 0.000 | 0.013 | 0.000 | 0.037 | 0.000 |
| Cesarean section | 0.001 | 0.598 | -0.006 | 0.000 | 0.011 | 0.000 | -0.038 | 0.000 | 0.016 | 0.000 | 0.008 | 0.000 |
| Other pregnancy/delivery complication | 0.015 | 0.000 | 0.003 | 0.000 | 0.006 | 0.000 | 0.000 | 0.795 | 0.005 | 0.000 | 0.027 | 0.000 |
| Mental/behavioral health diagnosis | 0.075 | 0.000 | 0.000 | 0.648 | -0.013 | 0.000 | 0.008 | 0.000 | 0.261 | 0.000 | 0.053 | 0.000 |
| Expansion state | 0.050 | 0.000 | 0.009 | 0.000 | 0.067 | 0.000 | 0.022 | 0.000 | -0.012 | 0.000 | 0.055 | 0.000 |
| Constant | 0.722 | 0.000 | 0.032 | 0.000 | 0.159 | 0.000 | 0.392 | 0.000 | 0.078 | 0.000 | 0.541 | 0.000 |

Notes: Estimates come from ordinary-least squares regression models where the outcome variable for each model is a binary indicator for having at least one visit between 61 days and 12 months postpartum. Sample includes enrollees with Medicaid-covered births in 2018 (see Appendix 1 for details). State Medicaid expansion status is as of January 2018. Ref= reference category. ^+^Reference category for urban vs. rural also includes 4,762 enrollees with an unknown urban/rural status due to missing data on the Rural-Urban Commuting Area code.

PANEL B: Enrollees with continuous 12-months enrollment in states with high-quality race/ethnicity data (N=423,674), controlling for race/ethnicity

|  | Any visit | | Postpartum evaluation | | Preventive/well | | Contraceptive management | | Mental/behavioral health care | | Acute or chronic illness | |
| --- | --- | --- | --- | --- | --- | --- | --- | --- | --- | --- | --- | --- |
|  | Coefficient | P-value | Coefficient | P-value | Coefficient | P-value | Coefficient | P-value | Coefficient | P-value | Coefficient | P-value |
| Age in years (ref= less than 19) |  |  |  |  |  |  |  |  |  |  |  |  |
| 19 to 24 | -0.040 | 0.000 | 0.000 | 0.998 | -0.024 | 0.000 | -0.063 | 0.000 | 0.008 | 0.007 | -0.029 | 0.000 |
| 25 to 29 | -0.043 | 0.000 | -0.002 | 0.314 | -0.018 | 0.000 | -0.108 | 0.000 | 0.025 | 0.000 | -0.024 | 0.000 |
| 30 to 34 | -0.048 | 0.000 | -0.001 | 0.745 | -0.014 | 0.000 | -0.141 | 0.000 | 0.024 | 0.000 | -0.018 | 0.000 |
| 35+ | -0.048 | 0.000 | 0.000 | 0.830 | -0.006 | 0.066 | -0.179 | 0.000 | 0.017 | 0.000 | -0.003 | 0.471 |
| Race/Ethnicity  (ref= white) |  |  |  |  |  |  |  |  |  |  |  |  |
| Hispanic | -0.029 | 0.000 | 0.005 | 0.000 | -0.005 | 0.003 | 0.056 | 0.000 | -0.118 | 0.000 | -0.030 | 0.000 |
| Black | -0.030 | 0.000 | 0.003 | 0.000 | 0.022 | 0.000 | 0.028 | 0.000 | -0.125 | 0.000 | -0.048 | 0.000 |
| All other | -0.024 | 0.000 | 0.002 | 0.196 | -0.005 | 0.049 | 0.015 | 0.000 | -0.128 | 0.000 | -0.015 | 0.000 |
| Missing | -0.020 | 0.000 | 0.006 | 0.000 | 0.020 | 0.000 | 0.002 | 0.540 | -0.076 | 0.000 | -0.010 | 0.004 |
| Rurality  (ref= urban^+^) |  |  |  |  |  |  |  |  |  |  |  |  |
| Rural residence | 0.040 | 0.000 | -0.003 | 0.000 | -0.030 | 0.000 | 0.002 | 0.215 | 0.034 | 0.000 | 0.071 | 0.000 |
| Eligibility status 12 months after birth  (ref= low-income parent) |  |  |  |  |  |  |  |  |  |  |  |  |
| Expansion adult | -0.031 | 0.000 | -0.001 | 0.060 | -0.015 | 0.000 | -0.034 | 0.000 | -0.008 | 0.000 | -0.025 | 0.000 |
| Pregnancy | -0.029 | 0.000 | 0.044 | 0.000 | -0.062 | 0.000 | -0.167 | 0.000 | -0.039 | 0.000 | 0.056 | 0.000 |
| Transitional Medical Assistance | 0.024 | 0.000 | -0.003 | 0.031 | 0.018 | 0.000 | 0.000 | 0.960 | 0.003 | 0.145 | 0.034 | 0.000 |
| Child | 0.000 | 0.998 | -0.006 | 0.002 | 0.037 | 0.000 | 0.012 | 0.012 | 0.011 | 0.004 | -0.006 | 0.231 |
| SSI | 0.050 | 0.000 | -0.003 | 0.087 | 0.022 | 0.000 | 0.005 | 0.298 | 0.112 | 0.000 | 0.056 | 0.000 |
| Other | -0.016 | 0.026 | 0.002 | 0.547 | 0.004 | 0.572 | -0.030 | 0.000 | 0.019 | 0.002 | 0.018 | 0.027 |
| Missing | -0.093 | 0.000 | -0.006 | 0.001 | -0.027 | 0.000 | -0.028 | 0.000 | -0.045 | 0.000 | -0.103 | 0.000 |
| Preexisting chronic condition | 0.041 | 0.000 | 0.000 | 0.521 | 0.012 | 0.000 | 0.002 | 0.145 | 0.012 | 0.000 | 0.067 | 0.000 |
| Preterm birth | 0.026 | 0.000 | 0.007 | 0.000 | 0.007 | 0.000 | 0.015 | 0.000 | 0.018 | 0.000 | 0.036 | 0.000 |
| Cesarean section | -0.001 | 0.446 | -0.006 | 0.000 | 0.009 | 0.000 | -0.041 | 0.000 | 0.014 | 0.000 | 0.007 | 0.000 |
| Other pregnancy/delivery complication | 0.016 | 0.000 | 0.004 | 0.000 | 0.003 | 0.073 | -0.001 | 0.602 | 0.006 | 0.000 | 0.028 | 0.000 |
| Mental/behavioral health diagnosis | 0.072 | 0.000 | 0.001 | 0.177 | -0.005 | 0.000 | 0.014 | 0.000 | 0.244 | 0.000 | 0.047 | 0.000 |
| Expansion state | 0.012 | 0.000 | 0.005 | 0.000 | 0.045 | 0.000 | -0.008 | 0.000 | -0.021 | 0.000 | 0.019 | 0.000 |
| Constant | 0.775 | 0.000 | 0.032 | 0.000 | 0.170 | 0.000 | 0.393 | 0.000 | 0.175 | 0.000 | 0.593 | 0.000 |

Notes: Estimates come from ordinary-least squares regression models where the outcome variable for each model is a binary indicator for having at least one visit between 61 days and 12 months postpartum. Sample includes enrollees with Medicaid-covered births in 2018 living in states with high-quality data on race/ethnicity (see Appendix 1 and Appendix 4 for details). State Medicaid expansion status is as of January 2018. Ref= reference category. ^+^Reference category for urban vs. rural also includes 2,131 enrollees with an unknown urban/rural status due to missing data on the Rural-Urban Commuting Area code.

PANEL C: Enrollees with continuous 12-months enrollment in all study states (N= 770,815), controlling for state

|  | Any visit, AL as reference state | | Any visit, AR as reference state | |
| --- | --- | --- | --- | --- |
|  | Coefficient | P-value | Coefficient | P-value |
| Age in years (ref= less than 19) |  |  |  |  |
| 19 to 24 | -0.033 | 0.000 | -0.033 | 0.000 |
| 25 to 29 | -0.038 | 0.000 | -0.038 | 0.000 |
| 30 to 34 | -0.041 | 0.000 | -0.041 | 0.000 |
| 35+ | -0.040 | 0.000 | -0.040 | 0.000 |
| Rurality (ref= urban^+^) |  |  |  |  |
| Rural residence | 0.044 | 0.000 | 0.044 | 0.000 |
| Eligibility status 12 months after birth (ref= low-income parent) |  |  |  |  |
| Expansion adult | -0.017 | 0.000 | -0.017 | 0.000 |
| Pregnancy | 0.000 | 0.879 | 0.000 | 0.879 |
| Transitional Medical Assistance | 0.021 | 0.000 | 0.021 | 0.000 |
| Child | 0.029 | 0.000 | 0.029 | 0.000 |
| SSI | 0.059 | 0.000 | 0.059 | 0.000 |
| Other | -0.003 | 0.384 | -0.003 | 0.384 |
| Missing | -0.070 | 0.000 | -0.070 | 0.000 |
| Preexisting chronic condition | 0.035 | 0.000 | 0.035 | 0.000 |
| Preterm birth | 0.029 | 0.000 | 0.029 | 0.000 |
| Cesarean section | 0.002 | 0.169 | 0.002 | 0.169 |
| Other pregnancy/delivery complication | 0.015 | 0.000 | 0.015 | 0.000 |
| Mental/behavioral health diagnosis | 0.070 | 0.000 | 0.070 | 0.000 |
| AL | Ref |  | -0.116 |  |
| AK | 0.234 | 0.000 | 0.118 | 0.000 |
| AZ | 0.224 | 0.000 | 0.108 | 0.000 |
| AR | 0.116 | 0.000 | Ref |  |
| CA | 0.233 | 0.000 | 0.118 | 0.000 |
| CO | 0.237 | 0.000 | 0.121 | 0.000 |
| CT | 0.372 | 0.000 | 0.257 | 0.000 |
| DC | 0.260 | 0.000 | 0.144 | 0.000 |
| DE | 0.287 | 0.000 | 0.171 | 0.000 |
| GA | 0.153 | 0.000 | 0.038 | 0.000 |
| HI | 0.187 | 0.000 | 0.071 | 0.000 |
| IA | 0.327 | 0.000 | 0.211 | 0.000 |
| ID | 0.217 | 0.000 | 0.101 | 0.000 |
| IL | 0.233 | 0.000 | 0.118 | 0.000 |
| IN | 0.205 | 0.000 | 0.090 | 0.000 |
| KS | 0.246 | 0.000 | 0.130 | 0.000 |
| KY | 0.251 | 0.000 | 0.135 | 0.000 |
| LA | 0.270 | 0.000 | 0.155 | 0.000 |
| MD | 0.326 | 0.000 | 0.210 | 0.000 |
| ME | 0.321 | 0.000 | 0.205 | 0.000 |
| MI | 0.234 | 0.000 | 0.119 | 0.000 |
| MO | 0.179 | 0.000 | 0.064 | 0.000 |
| MS | 0.309 | 0.000 | 0.194 | 0.000 |
| MT | 0.298 | 0.000 | 0.182 | 0.000 |
| NC | 0.218 | 0.000 | 0.103 | 0.000 |
| ND | 0.288 | 0.000 | 0.172 | 0.000 |
| NE | 0.314 | 0.000 | 0.199 | 0.000 |
| NH | 0.267 | 0.000 | 0.151 | 0.000 |
| NM | 0.271 | 0.000 | 0.156 | 0.000 |
| NV | 0.214 | 0.000 | 0.098 | 0.000 |
| NY | 0.340 | 0.000 | 0.224 | 0.000 |
| OH | 0.297 | 0.000 | 0.182 | 0.000 |
| OK | 0.212 | 0.000 | 0.096 | 0.000 |
| OR | 0.254 | 0.000 | 0.138 | 0.000 |
| PA | 0.291 | 0.000 | 0.175 | 0.000 |
| SC | 0.202 | 0.000 | 0.086 | 0.000 |
| SD | 0.266 | 0.000 | 0.150 | 0.000 |
| TN | 0.265 | 0.000 | 0.150 | 0.000 |
| TX | 0.221 | 0.000 | 0.105 | 0.000 |
| UT | 0.180 | 0.000 | 0.064 | 0.000 |
| VA | 0.144 | 0.000 | 0.028 | 0.000 |
| VT | 0.362 | 0.000 | 0.247 | 0.000 |
| WA | 0.297 | 0.000 | 0.181 | 0.000 |
| WI | 0.316 | 0.000 | 0.201 | 0.000 |
| WV | 0.140 | 0.000 | 0.024 | 0.001 |
| WY | 0.297 | 0.000 | 0.181 | 0.000 |
| constant | 0.501 | 0.000 | 0.616 | 0.000 |

Notes: Estimates come from ordinary-least squares regression models where the outcome variable for each model is a binary indicator for having at least one visit between 61 days and 12 months postpartum. Sample includes enrollees with Medicaid-covered births in 2018 (see Appendix 1 for details). State Medicaid expansion status is as of January 2018. Ref= reference category. ^+^Reference category for urban vs. rural also includes 4,762 enrollees with an unknown urban/rural status due to missing data on the Rural-Urban Commuting Area code.

## Appendix 6: Share of Medicaid enrollees with visits within 60 days and between 61 days and 12 months postpartum by individual characteristics, limited to expansion-eligible enrollees in ACA expansion states, 2018

|  | Births with 12 months continuous eligibility postpartum,  Expansion enrollees in expansion states | | | | | |
| --- | --- | --- | --- | --- | --- | --- |
|  | Visit within 60 days | | | Visit between 61 days and 12 months | | |
|  | N | Share | | N | Share | |
| All | 180,769 | 76.4% |  | 180,769 | 75.3% |  |
| Age in years |  |  |  |  |  |  |
| Less than 19^ | 4,980 | 74.8% |  | 4,980 | 77.3% |  |
| 19-24 | 59,633 | 74.2% |  | 59,633 | 74.8% | *** |
| 25-29 | 57,637 | 76.6% | *** | 57,637 | 75.3% | *** |
| 30-34 | 36,686 | 78.3% | *** | 36,686 | 75.6% | *** |
| 35 plus | 21,833 | 78.9% | *** | 21,833 | 75.7% | ** |
| Race/Ethnicity^+^ |  |  |  |  |  |  |
| White NH^ | 54,760 | 76.7% |  | 54,760 | 76.5% |  |
| Hispanic | 26,474 | 78.7% | *** | 26,474 | 72.8% | *** |
| Black NH | 21,517 | 73.1% | *** | 21,517 | 71.6% | *** |
| All other race/ethnicity groups | 7,105 | 80.8% | *** | 7,105 | 73.3% | *** |
| Missing race/ethnicity | 5,489 | 78.6% | *** | 5,489 | 71.4% | *** |
| State does not have sufficient race/ethnicity data quality | 65,424 | 75.6% | *** | 65,424 | 77.0% | ** |
| Urban/Rural residence |  |  |  |  |  |  |
| Urban^ | 143,565 | 76.7% |  | 143,565 | 74.5% |  |
| Rural | 36,117 | 75.1% | *** | 36,117 | 78.2% | *** |
| Condition During Pregnancy/Birth |  |  |  |  |  |  |
| No condition^ | 58,600 | 72.3% |  | 58,600 | 71.3% |  |
| Preexisting chronic condition | 40,244 | 81.2% | *** | 40,244 | 79.2% | *** |
| Preterm birth | 24,882 | 77.2% | *** | 24,882 | 78.3% | *** |
| Cesarean Section | 49,669 | 80.8% | *** | 49,669 | 76.6% | *** |
| Pregnancy or delivery complication | 43,946 | 80.2% | *** | 43,946 | 77.2% | *** |
| Mental or behavioral health diagnosis | 48,695 | 77.5% | *** | 48,695 | 80.6% | *** |

Source: 2018-2019 Transformed Medicaid Statistical Information System Analytic Files from 45 states plus DC.

Notes: Sample includes enrollees with Medicaid-covered births in 2018 living in states that had adopted ACA expansions and who were eligible for Medicaid through the ACA expansion. ^+^Race/ethnicity comparisons are limited states with sufficient race/ethnicity data quality to identify non-Hispanic white, non-Hispanic Black, and Hispanic enrollees. State Medicaid expansion status is as of January 2018. NH= non-Hispanic. SSI= Supplemental Security Income. */**/*** Estimate differs significantly from reference group (^) at the 0.10/0.05/0.01 level, using two-tailed tests.

## Appendix 7: Share of postpartum enrollees with postpartum evaluation visits within 60 days postpartum and between 61 days and 12 months postpartum, by state frequency of bundled payment codes for prenatal care, 2018

| Sample | Postpartum evaluation within 60 days postpartum | Postpartum evaluation between 61 days and 12 months postpartum |
| --- | --- | --- |
| All study states (N= 46 states) | 53.4% | 3.6% |
| States where > 5% and <20% of deliveries use bundled payment codes (N= 25 states) | 62.6% | 4.2% |
| States where > 20% of deliveries use bundled payment codes (N= 5 states) | 38.8% | 2.8% |
| All other study states (N= 16 states) | 58.1% | 3.9% |

SOURCE: Authors’ analysis of 2018-2019 Transformed Medicaid Statistical Information System Analytic Files from 45 states plus the District of Columbia and <https://www.medicaid.gov/dq-atlas/landing/topics/single/map?topic=g8m101&tafVersionId=23>.

Notes: Sample includes enrollees with Medicaid-covered births in 2018 (see Appendix 1 for details).

## Appendix 8: Share of Medicaid enrollees with visits within 60 days postpartum and between 61 days and 12 months postpartum, by individual characteristics, September-December 2018 births

|  | All births between September-December 2018 | | | Births between September-December 2018 with 12 months continuous eligibility postpartum | | | | | | | | |
| --- | --- | --- | --- | --- | --- | --- | --- | --- | --- | --- | --- | --- |
|  | Visit within 60 days | | | Visit between 61 days and 12 months | | | | | | | | |
|  | All states in sample | | | All states in sample | | | Expansion states | | | Non-expansion states | | |
|  | N | Share (%) | | N | Share (%) | | N | Share (%) | | N | Share (%) | |
| All | 479,429 | 67.0 |  | 252,376 | 76.6 |  | 195,197 | 77.5 |  | 57,179 | 73.5 |  |
| Age in years |  |  |  |  |  |  |  |  |  |  |  |  |
| Less than 19^ | 23,359 | 69.4 |  | 15,434 | 79.3 |  | 9,601 | 79.9 |  | 5,833 | 78.3 |  |
| 19-24 | 161,216 | 66.5 | *** | 85,042 | 76.5 | *** | 64,500 | 77.5 | *** | 20,542 | 73.4 | *** |
| 25-29 | 147,469 | 68.0 | *** | 77,544 | 76.2 | *** | 60,734 | 77.3 | *** | 16,810 | 72.3 | *** |
| 30-34 | 92,529 | 66.8 | *** | 47,344 | 76.5 | *** | 38,111 | 77.3 | *** | 9,233 | 73.0 | *** |
| 35 plus | 54,856 | 64.9 | *** | 27,012 | 76.4 | *** | 22,251 | 77.2 | *** | 4,761 | 72.9 | *** |
| Race/Ethnicity^+^ |  |  |  |  |  |  |  |  |  |  |  |  |
| White NH^ | 93,664 | 73.3 |  | 54,260 | 79.8 |  | 45,616 | 79.8 |  | 8,644 | 80.1 |  |
| Hispanic | 105,262 | 65.8 | *** | 40,126 | 74.2 | *** | 35,300 | 74.0 | *** | 4,826 | 75.5 | *** |
| Black NH | 50,820 | 71.2 | *** | 29,142 | 75.4 | *** | 23,649 | 75.5 | *** | 5,493 | 74.9 | *** |
| All other race/ethnicity groups | 17,250 | 71.8 | *** | 8,748 | 73.8 | *** | 7,110 | 73.3 | *** | 1,638 | 76.1 | *** |
| Missing race/ethnicity | 15,188 | 67.9 | *** | 7,104 | 76.3 | *** | 6,266 | 75.7 | *** | 838 | 80.9 |  |
| State does not have sufficient race/ethnicity data quality | 197,245 | 63.0 | *** | 112,996 | 76.4 | *** | 77,256 | 78.8 | *** | 35,740 | 71.1 | *** |
| Urban/Rural residence |  |  |  |  |  |  |  |  |  |  |  |  |
| Urban^ | 381,747 | 66.9 |  | 201,066 | 75.7 |  | 159,424 | 76.7 |  | 41,642 | 71.8 |  |
| Rural | 91,250 | 67.4 | *** | 49,091 | 80.0 | *** | 33,677 | 80.8 | *** | 15,414 | 78.3 | *** |
| Eligibility Status 12 months after birth |  |  |  |  |  |  |  |  |  |  |  |  |
| Parent^ |  |  |  | 134,050 | 77.0 |  | 100,301 | 78.3 |  | 33,749 | 73.1 |  |
| Adult Expansion^a^ |  |  |  | 58,266 | 76.0 | *** | 58,078 | 76.0 | *** | 188 | 76.6 |  |
| Pregnancy |  |  |  | 12,953 | 75.7 | *** | 8,378 | 76.0 | *** | 4,575 | 75.3 | *** |
| Transitional Medical Assistance |  |  |  | 15,514 | 78.8 | *** | 11,770 | 79.9 | *** | 3,744 | 75.6 | *** |
| Child |  |  |  | 9,697 | 79.1 | *** | 6,277 | 79.5 | ** | 3,420 | 78.3 | *** |
| SSI |  |  |  | 5,529 | 85.3 | *** | 3,541 | 86.0 | *** | 1,988 | 84.1 | *** |
| Other Eligibility Status |  |  |  | 5,023 | 70.5 | *** | 1,104 | 80.7 | * | 3,919 | 67.6 | *** |
| Missing or Multiple Eligibility Status |  |  |  | 11,344 | 69.0 | *** | 5,748 | 67.8 | *** | 5,596 | 70.3 | *** |
| Condition During Pregnancy/Birth |  |  |  |  |  |  |  |  |  |  |  |  |
| No condition^ | 153,633 | 60.0 |  | 71,302 | 71.7 |  | 55,411 | 72.7 |  | 15,891 | 68.4 |  |
| Preexisting chronic condition | 112,453 | 72.9 | *** | 63,159 | 80.0 | *** | 49,288 | 80.9 | *** | 13,871 | 77.0 | *** |
| Preterm birth | 69,596 | 70.7 | *** | 40,203 | 79.4 | *** | 30,325 | 80.3 | *** | 9,878 | 76.7 | *** |
| Cesarean Section | 130,583 | 72.2 | *** | 69,435 | 77.3 | *** | 53,653 | 78.3 | *** | 15,782 | 73.9 | *** |
| Pregnancy or delivery complication | 118,360 | 71.0 | *** | 62,928 | 78.7 | *** | 48,825 | 79.6 | *** | 14,103 | 75.6 | *** |
| Mental or behavioral health diagnosis | 123,431 | 74.0 | *** | 82,885 | 82.6 | *** | 63,632 | 83.4 | *** | 19,253 | 79.9 | *** |

Source: 2018-2019 Transformed Medicaid Statistical Information System Analytic Files from 45 states plus DC.

Notes: Sample includes enrollees with Medicaid-covered births in September-December 2018 (see Appendix 1 for details). ^+^Race/ethnicity comparisons are limited to the 20 states with sufficient race/ethnicity data quality (see Appendix 4). State Medicaid expansion status is as of January 2018. AIAN= American Indian Alaskan Native. NH= non-Hispanic. SSI= Supplemental Security Income. */**/*** Estimate differs significantly from reference group (^) at the 0.10/0.05/0.01 level, using two-tailed tests. ^a^Since we classified expansion status as of January 2018, a small number of enrollees from “nonexpansion” states that implemented Medicaid expansion during the study period had an eligibility pathway of “adult expansion” at 12 months postpartum.

## Appendix 9: Share of Medicaid enrollees with at least one outpatient visit within the full 12-month postpartum period, among those with continuous enrollment for 12 months, 2018 deliveries (N= 770,815)

Source: 2018-2019 Transformed Medicaid Statistical Information System Analytic Files from 45 states plus DC.

Notes: Sample includes enrollees with Medicaid-covered births in 2018 with continuous enrollment for the 12-month postpartum period (see Appendix 1 for details). Shares by type of care sum to more than the share with any visit because enrollees may have more than one visit and a small number (3.3%) of visits include more than one type of care. State Medicaid expansion status is as of January 2018. *** Estimate differs significantly between expansion and non-expansion states at the 0.01 level, using two-tailed tests. Eval.= evaluation. Mgmt=management. BH =behavioral health.

## Appendix 10: Share of Medicaid enrollees with at least one outpatient visit within 60 days postpartum, among those with continuous enrollment for 12 months, 2018 (N= 770,815)

Source: 2018-2019 Transformed Medicaid Statistical Information System Analytic Files from 45 states plus DC.

Notes: Sample includes enrollees with Medicaid-covered births in 2018 with continuous enrollment for the 12-month postpartum period (see Appendix 1 for details).Shares by type of care sum to more than the share with any visit because enrollees may have more than one visit and a small number (3.3%) of visits include more than one type of care. State Medicaid expansion status is as of January 2018. *** Estimate differs significantly between expansion and non-expansion states at the 0.01 level, using two-tailed tests. Eval.= evaluation. Mgmt=management. BH =behavioral health.

## Appendix 11: Share of Medicaid enrollees with visits during the full 12-month postpartum period by individual characteristics, 2018

|  | All births in sample | | | Births with 12 months continuous eligibility postpartum | | | | | | | | |
| --- | --- | --- | --- | --- | --- | --- | --- | --- | --- | --- | --- | --- |
|  | Visit within 12 months | | | Visit within 12 months | | | | | | | | |
|  |  |  |  | All states in sample | | | Expansion states | | | Non-expansion states | | |
|  | N | Share | | N | Share | | N | Share | | N | Share | |
| All | 1,453,767 | 78.10% |  | 770,815 | 90.60% |  | 597,799 | 91.20% |  | 173,016 | 88.40% |  |
| Age in years |  |  |  |  |  |  |  |  |  |  |  |  |
| Less than 19^ | 72,084 | 83.40% |  | 47,834 | 91.30% |  | 29,868 | 91.60% |  | 17,966 | 90.70% |  |
| 19-24 | 492,196 | 79.00% | *** | 262,905 | 89.70% | *** | 200,176 | 90.30% | *** | 62,729 | 87.90% | *** |
| 25-29 | 447,825 | 79.00% | *** | 237,006 | 90.80% | *** | 186,059 | 91.50% |  | 50,947 | 88.10% | *** |
| 30-34 | 278,484 | 76.50% | *** | 142,748 | 91.20% |  | 115,163 | 91.80% |  | 27,585 | 88.40% | *** |
| 35 plus | 163,178 | 73.80% | *** | 80,322 | 91.50% |  | 66,533 | 92.00% | ** | 13,789 | 88.80% | *** |
| Race/Ethnicity^+^ |  |  |  |  |  |  |  |  |  |  |  |  |
| White NH^ | 286,441 | 84.30% |  | 166,731 | 92.30% |  | 140,459 | 92.40% |  | 26,272 | 91.70% |  |
| Hispanic | 312,545 | 72.70% | *** | 120,409 | 92.20% |  | 105,666 | 92.30% |  | 14,743 | 91.40% |  |
| Black NH | 153,342 | 83.20% | *** | 89,077 | 90.20% | *** | 72,112 | 90.40% | *** | 16,965 | 89.20% | *** |
| All other race/ethnicity groups | 51,905 | 81.30% | *** | 26,340 | 92.00% |  | 21,429 | 92.60% |  | 4,911 | 89.20% | *** |
| Missing race/ethnicity | 44,833 | 76.10% | *** | 21,117 | 91.80% | * | 18,573 | 92.00% | * | 2,544 | 90.90% |  |
| State does not have sufficient race/ethnicity data quality | 604,701 | 76.60% | *** | 347,141 | 89.20% | *** | 239,560 | 90.20% | *** | 107,581 | 86.90% | *** |
| Urban/Rural residence | |  |  |  |  |  |  |  |  |  |  |  |
| Urban^ | 1,156,957 | 77.70% |  | 614,434 | 90.40% |  | 488,359 | 91.10% |  | 126,075 | 87.60% |  |
| Rural | 279,314 | 80.20% | *** | 151,619 | 91.40% | *** | 104,877 | 91.80% | *** | 46,742 | 90.60% | *** |
| Eligibility Status 12 months after birth | | |  |  |  |  |  |  |  |  |  |  |
| Parent^ |  |  |  | 407,980 | 91.00% |  | 305,977 | 92.00% |  | 102,003 | 87.90% |  |
| Adult Expansion^a^ |  |  |  | 181,146 | 90.40% | *** | 180,769 | 90.40% | *** | 377 | 88.90% |  |
| Pregnancy |  |  |  | 39,893 | 89.60% | *** | 23,924 | 89.60% | *** | 15,969 | 89.60% | *** |
| Transitional Medical Assistance | |  |  | 46,014 | 92.00% | *** | 34,777 | 92.30% |  | 11,237 | 91.10% | *** |
| Child |  |  |  | 30,881 | 90.30% | *** | 20,592 | 89.80% | *** | 10,289 | 91.10% | *** |
| SSI |  |  |  | 17,068 | 92.60% | *** | 11,072 | 93.30% | *** | 5,996 | 91.40% | *** |
| Other Eligibility Status | |  |  | 12,995 | 86.70% | *** | 3,260 | 93.30% | ** | 9,735 | 84.50% | *** |
| Missing or Multiple Eligibility Status | | |  | 34,838 | 87.40% | *** | 17,428 | 86.60% | *** | 17,410 | 88.20% |  |
| Condition During Pregnancy/Birth |  |  |  |  |  |  |  |  |  |  |  |  |
| No condition^ | 498,384 | 72.40% |  | 238,948 | 88.20% |  | 186,028 | 88.90% |  | 52,920 | 85.60% |  |
| Preexisting chronic condition | 318,860 | 82.90% | *** | 179,353 | 93.10% | *** | 140,540 | 93.60% | *** | 38,813 | 91.00% | *** |
| Preterm birth | 202,023 | 82.60% | *** | 116,803 | 91.50% | *** | 87,943 | 92.10% | *** | 28,860 | 89.70% | *** |
| Cesarean Section | 398,392 | 81.50% | *** | 213,193 | 92.20% | *** | 164,803 | 92.90% | *** | 48,390 | 89.70% | *** |
| Pregnancy or delivery complication | 348,182 | 80.50% | *** | 186,121 | 92.30% | *** | 144,924 | 92.90% | *** | 41,197 | 90.10% | *** |
| Mental or behavioral health diagnosis | 325,379 | 86.90% | *** | 218,781 | 92.40% | *** | 169,436 | 92.80% | *** | 49,345 | 91.00% | *** |

Source: 2018-2019 Transformed Medicaid Statistical Information System Analytic Files from 45 states plus DC.

Notes: Sample includes enrollees with Medicaid-covered births in 2018 (see Appendix 1 for details). ^+^Race/ethnicity comparisons are limited to the 20 states with sufficient race/ethnicity data quality to identify non-Hispanic white, non-Hispanic Black, and Hispanic enrollees. Analyses of other race/ethnicity groups for states with sufficient data quality are provided in Appendix 4. State Medicaid expansion status is as of January 2018. NH= non-Hispanic. SSI= Supplemental Security Income. */**/*** Estimate differs significantly from reference group (^) at the 0.10/0.05/0.01 level, using two-tailed tests. ^a^Since we classified expansion status as of January 2018, a small number of enrollees from “nonexpansion” states that implemented Medicaid expansion during the study period had an eligibility pathway of “adult expansion” at 12 months postpartum.
